# Real-time, model-based magnetic field correction for moving, wearable MEG

**DOI:** 10.1016/j.neuroimage.2023.120252

**Published:** 2023-09

**Authors:** Stephanie Mellor, Tim M. Tierney, Robert A. Seymour, Ryan C. Timms, George C. O'Neill, Nicholas Alexander, Meaghan E. Spedden, Heather Payne, Gareth R. Barnes

**Affiliations:** Wellcome Centre for Human Neuroimaging, UCL Queen Square Institute of Neurology, University College London, London WC1N 3AR, UK

**Keywords:** Magnetoencephalography, MEG, Optically pumped magnetometer, Magnetic field correction, Walking OP-MEG, Auditory evoked field

## Abstract

Most neuroimaging techniques require the participant to remain still for reliable recordings to be made. Optically pumped magnetometer (OPM) based magnetoencephalography (OP-MEG) however, is a neuroimaging technique which can be used to measure neural signals during large participant movement (approximately 1 m) within a magnetically shielded room (MSR) (Boto et al., 2018; Seymour et al., 2021). Nevertheless, environmental magnetic fields vary both spatially and temporally and OPMs can only operate within a limited magnetic field range, which constrains participant movement. Here we implement real-time updates to electromagnetic coils mounted on-board of the OPMs, to cancel out the changing background magnetic fields. The coil currents were chosen based on a continually updating harmonic model of the background magnetic field, effectively implementing homogeneous field correction (HFC) in real-time (Tierney et al., 2021). During a stationary, empty room recording, we show an improvement in very low frequency noise of 24 dB. In an auditory paradigm, during participant movement of up to 2 m within a magnetically shielded room, introduction of the real-time correction more than doubled the proportion of trials in which no sensor saturated recorded outside of a 50 cm radius from the optimally-shielded centre of the room. The main advantage of such model-based (rather than direct) feedback is that it could allow one to correct field components along unmeasured OPM axes, potentially mitigating sensor gain and calibration issues (Borna et al., 2022).

## Highlights

• Zero-field OPMs operate within a limited magnetic field range.
• We correct for background field changes in real-time using coils on-board the OPMs.
• We used a model of the background field based on low-order regular solid harmonics.
• We were able to record auditory evoked fields during movements of 1.5m - 2m.

## 1 Introduction

Magnetoencephalography (MEG) is a non-invasive functional neuroimaging technique with millimetre spatial and millisecond temporal resolution (Hämäläinen et al., 1993). Magnetic fields arising from neuronal current flows are measured and their source inferred (Baillet, 2017). However, the magnitude of these fields is on the order of 10s-100s of femto-Tesla and so highly sensitive magnetic sensors are needed to detect them.

Recently, optically pumped magnetometers (OPMs) have become a viable alternative sensor to traditional large and fixed superconducting sensor (SQUID) arrays (Johnson et al., 2010; Kim et al., 2014; Shah and Wakai, 2013; Xia et al., 2006). Systems can be built where OPMs are worn directly on the scalp, meaning that the participant can move around, in experiments previously considered inconceivable with SQUID-MEG (Boto et al., 2018; Hill et al., 2019; Holmes et al., 2021).

However, the range of movement possible during OP-MEG recordings is limited by the background magnetic field. Many commercial OPMs operate in the Spin Exchange Relaxation Free (SERF) regime (Happer and Tang, 1973; Shah et al., 2007), and as such have a limited operating range around zero-field. Fundamentally, for the sensors we typically use for OP-MEG, their absolute maximum range is limited to within ±10 nT, but their gain is non-linear and reduces with magnetic field strength, so increasing fields mean increasing gain errors (Iivanainen et al., 2019; Tierney et al., 2019). Additionally, magnetic fields along the laser propagation axis within the OPM lead to so-called cross-axis projection errors (CAPE), whereby the sensitive axis of the OPM is altered and amplitude and phase errors are introduced (Borna et al., 2022). Therefore, it is generally advisable that the magnetic field is kept below 2 nT over the course of an OP-MEG recording. No brain signal of interest will be this large, but the background magnetic field from the environment is several orders of magnitude larger than this value (Mellor et al., 2021).

Therefore, to allow OP-MEG recordings, these gain and saturation issues are generally minimised through use of magnetic shielding. Magnetically shielded rooms (MSRs), made of passive magnetic shielding such as mu-metal and aluminium, can bring the static (0 Hz) background magnetic field to below 10 nT. Active electromagnetic shielding may also be used (Holmes et al., 2018). Here, electromagnetic coils aim to generate a magnetic field which is equal and opposite to the background magnetic field, thus cancelling it out by the principle of superposition. The currents through these coils may either be constant or dynamically updated through an experiment to compensate for temporally changing background interference (Holmes et al., 2019).

Most, if not all, commercially available OPMs contain electromagnetic coils to cancel the magnetic field locally at the sensor (Osborne et al., 2018). These fields are commonly set at the beginning of a recording and not changed until it is finished, although dynamic, closed-loop systems are becoming more common (Lee et al., 2014; Nardelli et al., 2019; Robinson et al., 2022). These closed-loop systems currently either apply a current linearly proportional to the instantaneously measured magnetic field component on each OPM, or require additional reference sensors to record the interference field.

Here we propose an alternative dynamic feedback system, whereby a model of the background magnetic field is created from the recordings from a whole-head OP-MEG array. The background field at each OPM is then predicted and nulled at each sensor using the electromagnetic coils on-board each OPM. We model the background magnetic field as a homogeneous field across the scanner-cast at a given time-point. We update this model every 10 ms, meaning that the nulling can compensate for changes in the background magnetic field over time. In essence, we have implemented homogeneous field correction (HFC) (Tierney et al., 2021) in real-time.

The paper proceeds as follows. Firstly, we provide a brief introduction to the HFC model and how we have implemented it for our OP-MEG system. We then demonstrate this system with a stationary, empty helmet and look at its effectiveness in minimising temporal changes in magnetic field. We then look at its effectiveness in minimising movement related magnetic field changes in an auditory OP-MEG experiment with large (up to 2 m) participant movements and examine the auditory evoked responses with and without this feedback in place.

## 2 Methods

### 2.1 Homogeneous field correction

Homogeneous field correction is a method for modelling the background magnetic field during an MEG recording, which has previously been used successfully for reducing magnetic interference in OP-MEG data post-hoc (Hill et al., 2022; Seymour et al., 2022, 2021; Tierney et al., 2021). The main assumption within HFC is that, at a given time point, the background, interference magnetic field across the OPM array is described by a sum of the gradients of regular solid harmonics. In its simplest form (which we use here), this is equivalent to saying that at a given time point, the background magnetic field is spatially homogeneous across the OPM array, and can be described by only three parameters, $B_{\mathrm{background}}=\left[B_{x},B_{y},B_{z}\right]$. In the recordings presented here, we used an array of OPMs (Gen-2.0 QZFM, QuSpin; Louisville, CO), with two orthogonal recording axes, meaning that each OPM sensor had two channels, one radial to the head and one tangential to it. Under the homogeneous field model, the interference from the background field recorded by a given sensor channel, i, is

(1) $$y_{i}=B_{background}\cdot \rho_{i}=B_{x}\rho_{ix}+B_{y}\rho_{iy}+B_{z}\rho_{iz},$$

where $\rho_{i}$ is the orientation of sensor channel i. Considering an array of sensors, the background interference can then be described as

(2) $$Y=NB_{background},$$

where

$$N={\left[\begin{array}{cccc} \rho_{1} & \rho_{2} & \rho_{3} & \cdots \end{array}\right]}^{T},$$

(3) $$Y={\left[\begin{array}{cccc} y_{1} & y_{2} & y_{3} & \cdots \end{array}\right]}^{T}.$$

N and Y include all sensor channels. As such, $N\;\in R^{2N_{\text{OPMs}}\;\times \;3}$ and $Y\;\in R^{2N_{\text{OPMs}}\;\times \;1}$ where $N_{OPMs}$ is the number of recording OPMs. Then the background magnetic field can be estimated by the multiplication of Y with the Moore-Penrose pseudo-inverse of the sensor orientation matrix N, i.e. ${\left[B_{x},B_{y},B_{z}\right]}^{T}=N^{†}Y$.

The background interference at any orientation can then be estimated by the dot product between that orientation vector and the estimated magnetic field. To determine the desired feedback for our OPM array, we construct an orientation matrix M= ${\left[\begin{array}{cccc} \mu_{1} & \mu_{2} & \mu_{3} & \cdots \end{array}\right]}^{T}$ of orientations of the feedback coils ($\mu_{i}$). Since we have chosen to feedback via the electromagnetic coils on board the OPM and these coils are orientated in the same direction as the OPM recording channels, $M\in R^{2M_{OPMs}\;\times 3}$ (where $M_{OPMs}$ is the number of OPMs to feedback to) is the same matrix as N, except that it is made up of the orientations of the OPMs to feedback to, which are not necessarily the same as the channels from which the model was created. Then the desired feedback can be simply calculated as:

(4) $$\text{Desired}\;\text{Feedback}=MN^{†}Y.$$

The coordinate frame of the orientations and positions of the OPM recording channels and compensation coils can be chosen so that the projection matrix $MN^{†}$ is constant throughout the experiment, despite participant movement, as in our system the recording channels and compensation coils are held rigidly with respect to one another. As such, it can be calculated beforehand, minimising the time needed to compute the magnetic field model. We therefore chose to express $MN^{†}$ in the coordinate frame of the scanner-cast. To account for temporal changes in the background field, due both to temporal fluctuations and participant movement, the model ($\left[B_{x},B_{y},B_{z}\right]$) was recalculated throughout the experiment. In HFC applied post-hoc, the model is recalculated for each recorded datapoint.

### 2.2 Implementation

The feedback system was implemented in LabView 2017, with all computations done natively. The output from an array of dual-axis, zero-field magnetometers (Gen-2.0 QZFM, QuSpin; Louisville, CO) was recorded via a 16-bit analogue to digital converter (NI-9205, National Instruments; Austin, TX). The data were read into LabView in 60 sample chunks and were sampled at 6 kHz. As such, each chunk spanned 10 ms. To model the background magnetic field, each chunk of data was averaged over time (such that each chunk produced one average $Y\in R^{N_{OPMs}\times 1}$ vector) and a model created from the averaged data. Ideally, the HFC model would be created for each datapoint, but the average of 60 datapoints was used for two reasons. Firstly, it was computationally feasible to recalculate the model every 10 ms and was only possible to send a command to each OPM every 5 ms, far less than the 6 kHz at which we sampled. Secondly, it introduced a moving average filter (−3 dB point at 44 Hz), reducing aliasing from higher frequency signals than we could feedback at.

**Fig. 1:**
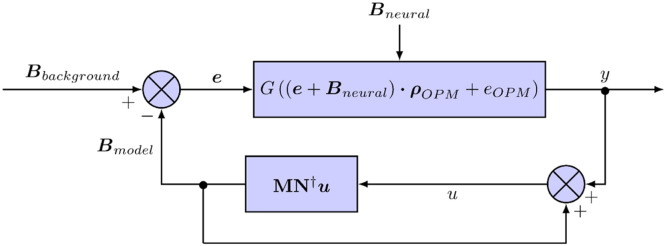
Control system employed to minimise the background field at the OPMs. We seek to minimise the error (e) between the background field ($B_{\mathrm{back}\mathrm{grou}\mathrm{nd}}$) and the feedback, modelled field ($B_{\mathrm{model}}$). The sensor recording, y, is determined by the OPM gain (G, unitless), the difference between the background field and the feedback field (e), the signal of interest ($B_{\mathrm{neur}\mathrm{al}}$), the sensitive axis orientation of the OPM ($\rho_{\mathrm{OPM}}$) and the sensor white noise ($e_{\mathrm{OPM}}$, determined by the OPM electronics and laser (Osborne et al., 2018)). A simplified signal equation is assumed here, although the full equation can be found in Iivanainen et al. (2019). The current OPM recording (y) is added to the previously fed-back magnetic field, in order to estimate the field component at the OPM which would be observed had no feedback been applied. HFC is then applied to this sum (u), through the matrix multiplication ${\mathrm{MN}}^{†}u$.The vector, u, is the column vector of the estimated fields (u) for each of the OPM channels. The matrix N is the orientation of all sensors ordered to correspond to the input from the OPMs, while M is also the orientation of the sensors but ordered to correspond to the digital output of the computer.

In a separate thread from that recording the OPMs, the modelled background field was applied to the sensitive axes of the sensors - named the Y and Z axes. The (unrecorded) X axis was left in its initialised state. The field was applied using the on-board OPM coils, which were controlled digitally via USB. Commands were sent to each sensitive axis every 10 ms. As it was only possible to send a serial command to the OPMs every 5 ms, the field was first updated on every Y axis, then after a 5 ms wait, the field on the Z axis was updated. The basic control algorithm is shown in Fig. 1.

We tested low-pass filtering both the model input and output in order to improve the feedback performance. As the base version of LabView does not provide an inbuilt point-by-point (i.e. real-time) multi-channel filter, a custom function was written. The type of filter was a fourth order Butterworth filter, comprised of two digital biquads, which are second order filters, so named because their transfer function in the z-domain is the ratio of two quadratic functions. The biquads were implemented in the transposed direct form II (Chowdhury, 2020; Smith, 2007).

### 2.3 Experimental data

**Fig. 2:**
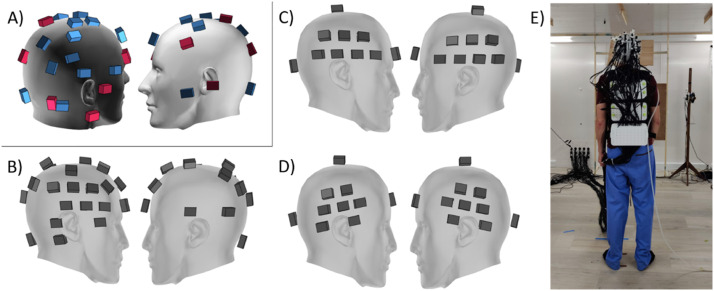
Positions of the OPMs used in each validation experiment in MNI space. The participant's head is represented by the grey mesh (https://www.turbosquid.com/3d-models/male-head-obj/346686). (A) Sensor positions during the environmental noise recordings. The pink OPMs had feedback applied, while the blue OPMs were used for reference measurement. (B-D) Sensor positions during the auditory OP-MEG experiment. (B) participant 1 initial recording, (C) participant 1 main recording and (D) participant 2. (E) Photograph of set up for auditory experiment, showing the backpack holding the cables and ear tube.

We performed two validation experiments in order to test our implementation of real-time HFC. Both were performed within the OPM MSR at UCL (Magnetic Shields Limited, 4-layer MSR, internal dimensions 3 m x 4 m x 2.2 m. Field within central cubic metre ∼2 nT after degaussing the inner mu-metal layer). Firstly, we set the array of OPMs onto a table in the centre of the room and recorded the environmental noise. We looked at the decrease in this noise when feedback was turned on for a subset of the OPMs, although the model was created from all recording channels. By recording the feedback on and off channels simultaneously, we could compare similar background noise conditions. Secondly, we recorded MEG from two healthy participants during an auditory evoked paradigm first while seated and then while walking around the room, both with and without feedback to all of the sensors. In both experiments, the OPMs were placed within a 3D printed scanner-cast of known geometry, in order to determine the sensor orientations relative to one another. The sensor arrays for each experiment are shown in Fig. 2. The sensors were operated in dual axis mode, meaning that they recorded the magnetic field radial to the scalp (Y channels) and along one axis tangential to it (Z channels), but the field component along the OPM laser axis (X axis) was unmeasured.

Prior to OPM recordings, the field at each OPM was minimised by degaussing the MSR (Altarev et al., 2015) and, to null the remnant background magnetic field locally, the currents through the on-sensor coils (the same coils which were used to feedback) were optimised according to the manufacturers’ procedures. During the OPM recordings, the intended feedback ($B_{model}$ in Fig. 1) was added to these initial field zeroing magnetic fields. The OPMs were then calibrated according to the manufacturer's procedure. This involves a magnetic field of known amplitude being applied to the sensor, again using the on-sensor coils. The output of the sensor is then measured and calibrated against the known field. This full procedure (degaussing, sensor field zeroing and calibration) was performed once for each set of experiments. I.e., the environmental noise recordings were taken one after another, first without any filter and then with one included, and the calibration was performed only before the first recording. Sensor field zeroing and calibration (without degaussing) was additionally performed during the auditory experiments after the participant stood up for the first time at the centre of the room, to optimise the OPM on-sensor coils for the participant's new position. The door to the MSR was not opened between blocks of the same experiment.

#### 2.3.1 Environmental noise recordings

In testing the performance of this feedback to compensate for environmental drifts, we first left the sensor array stationary in the centre of the room and recorded from the OPMs for 40 min. We turned the feedback on to a subset of the channels for the central 30 min of the recording. The channel positions are shown in Fig. 2. There were 21 recording OPMs (equating to 42 channels) and feedback was applied to 11 of them (22 channels). We performed this recording twice, first without any filtering and then with a 1 Hz low-pass filter to the intended feedback. A 1 Hz filter was chosen as the feedback increased the noise in the data above this frequency when no filter was used.

We evaluated the performance of the system by the median shielding factor, defined as follows:

(5) $$\text{mSF}=20\;\text{log}\left(\frac{\text{median}\left(\text{PS}D_{\text{feedback}\;\text{off}}\right)}{\text{median}\left(\text{PS}D_{\text{feedback}\;\text{on}}\right)}\right)$$

The power spectral density (PSD) was calculated according to Welch's method using 20 s chunks of data, only during the period where the feedback was turned on, and the median is taken over channels.

#### 2.3.2 Auditory evoked response recording

We recorded OP-MEG from two healthy adult participants (male, right-handed, 55-years old and 29-years old) during an auditory evoked paradigm. The participants sat in the centre of the MSR for the first 4 blocks (2 with feedback on, 2 with feedback off, randomly ordered) and walked around the MSR for the second set of 4 blocks (2 feedback on, 2 feedback off), while their position was recorded using an array of 6 OptiTrack Flex 13 cameras (Natural Point), which sampled their position at 120 Hz. The auditory stimulus was delivered binaurally via MEG compatible ear tubes. The participants provided written informed consent and the study was approved by the University College London Research Ethics Committee.

##### 2.3.2.1 Paradigm

The auditory evoked paradigm was the same as used by Seymour et al. (2021). It is a roving mismatch negativity task based on Garrido et al. (2008), meaning that a set of consecutive, identical tones was presented to the participant, followed by a set of a different frequency, the first of which is defined as the “deviant”. 70 ms long tones were presented (with 5 ms rise and fall times). The length of each set of consecutive tones varied between 1 and 11 across the experiment. This length was randomly chosen from a weighted distribution, with a 2.5% chance of 1 or 2 tones, 2.75% chance of 3 or 4 tones and 12.5% chance of 5–11 tones. The frequency of each set was randomly selected from 500 Hz, 550 Hz, 600 Hz, 650 Hz, 700 Hz, 750 Hz or 800 Hz with equal probability of each possible frequency. The inter-stimulus interval was 0.5 s and no stimulus jitter was included. 80 deviant tones were included in each recording block, which led to approximately 570 tones in each block. Each block was approximately 275–300 s long. We collapsed our analysis across all of the tones, rather than focussing on the difference between deviants and repeated tones. The volume of the stimuli was adjusted to be comfortable for the participant. The stimuli were presented via PsychoPy version 3.2.4 (Peirce, 2009).

##### 2.3.2.2 Acquisition

17 dual-axis OPMs (Gen-2.0 QZFM, QuSpin; Louisville, CO) were used, with feedback applied to all of them. They were held on the participants’ heads with bespoke, 3D printed scanner-casts, based on the participants’ MRIs (Meyer et al., 2017), simplifying co-registration between the MRI and sensor positions. The sensor positions were concentrated around the participants’ auditory cortices, while 3 OPMs were placed along the midline of the head to increase the spatial coverage. Six retro-reflective markers were also placed on each scanner-cast in order to track the scanner-cast's position and rotation. The participants were asked to walk around the room in a repeating pattern (shown in Fig. 5), including moving outside of the central 1 m^3^ where experiments usually occur. The gain of the OPM electronics was set such that the OPM channels saturated at approximately 1.5 nT (although this varies between OPMs). Eight blocks of the approximately 5-minute task were undertaken. Initially, the participants were seated with their head unconstrained (but without any instruction to move) for four blocks, during which feedback was applied for two of the four blocks. A 1 Hz low-pass filter was applied to the intended feedback field in order to minimise high frequency noise in the feedback. The participants then stood in the centre of the room, the OPMs were recalibrated, and during the next four blocks, the participants walked through the room. Again, during two walking blocks, no feedback was applied. Feedback was applied during the other two walking blocks, with a 1 Hz low-pass filter applied to the intended output field. The order of the blocks for participant 1 was feedback on, off, off, on, on, on, off, off and for participant 2 was off, on, off, on, on, on, off, off. The participants were unaware of whether feedback was included or not.

An earlier recording from participant 1 is also included (referred to as Participant 1, Initial). In this recording, only the walking condition was included. 5 OptiTrack Flex 13 cameras were used while the 6th simultaneously recorded video. The stimulus was delivered monaurally to the participant's right ear. 23 dual-axis OPMs were used but were placed asymmetrically. Five retroreflective markers were used to track the scanner-cast's position. The order of the blocks was feedback off, on, on, off and the participant was informed of the order of the first two blocks but was unaware of whether feedback was on or off for the second two.

To hold the weight of the OPM cables and ear tubes, the participants wore a backpack into which each OPM cable and ear tube clipped. To help the cables follow the participants as they moved around the room, the ear tubes were taped to a rotating stool. The participants also held the bundle of OPM cables (between the backpack and room wall) to avoid tripping whilst walking. This is shown in Fig. 2. The cables from the OPMs and ear tubes mechanically limited the participants’ movement to within approximately 2.5 m of where the cables entered the room.

The motion tracking and OPM recordings were synchronised by a 5 V pulse to an OPM trigger. From Matlab, on the same computer as the motion tracking was recorded, a command was first sent via the local network to the motion tracking software (Motive) to start the recording, using OptiTrack's NatNet SDK (https://docs.optitrack.com/developer-tools/natnet-sdk). Using the data acquisition toolbox in Matlab, a 5 V signal was then sent from a digital to analogue converter (NI USB-6009, National Instruments; Austin, TX), which was connected to the analogue to digital converter recording the OPM signals with a coaxial cable. At the end of the recording, a command was sent to Motive to stop the recording and to the digital to analogue converter to return to zero.

##### 2.3.2.3 Analysis

Analysis of the data was performed in Matlab 2021b, using SPM (Litvak et al., 2011) for the analysis and the FieldTrip Toolbox (Oostenveld et al., 2011) for the topographical representation of the data, as well as custom scripts to identify saturated periods in the OPM data and to synchronise it with the motion tracking recordings. The OPM data were pre-processed by first downsampling to 1 kHz for computational efficiency. The OPM data were then cropped to match the period when the motion tracking system was recording, and the motion tracking data were up-sampled to match this 1 kHz sampling rate.

We then determined the timepoints at which each OPM channel was saturated. The saturation point was approximately 1.5 nT but varied between sensors. We therefore, for each channel of the OPM data, discretised the data from the entire (approximately 5 minute) block by amplitude into bins of 1 pT width, ignoring any temporal information. We observed that the distribution of the measured data was approximately Gaussian. To determine if a channel had saturated, we looked for deviations from this distribution at the amplitude extremities of the data. If there were more than double the number of datapoints in the maximum (or minimum) 5 bins than the previous 5 bins, these maximum (or minimum) 5 bins were marked as saturated. This threshold of 5 bins (or equivalently 5 pT) was chosen empirically. When determining if a channel was saturated, an additional check was included so that no datapoints with a magnitude lower than 1 nT could be marked as saturated. This was only necessary in the seated recordings presented here, as then the data did not follow the same distribution as the walking recordings. We have made the code to test when the OPMs saturated publicly available (https://github.com/stephaniemellor/OPMEGfeedback).

At some timepoints, data were missing from the motion tracking recording, either due to occlusion of the retro-reflective markers or as a result of the participant stepping outside of the field of view of three or more cameras (a minimum of two cameras must observe the markers for their position to be reconstructed). This was particularly true at the edges of the MSR. Where 3 or more markers were present, gaps smaller than 8.3 s were filled within Motive (OptiTrack's software) using the so-called “model based” method, where the position of missing markers is estimated from their trajectories and the positions of present markers. Additionally, in the earlier recording from participant 1, a few (4 for block 3, 5 for blocks 2 and 4) positions at the front of the room were first estimated from a simultaneously recorded video and manually added to the motion recording. Remaining gaps were then interpolated in Matlab. For gaps of less than 0.2 s, this was done via linear interpolation. For larger gaps, this was done by shape-preserving piecewise cubic interpolation, using Matlab's fit function with the 'pchip' model option.

OPM data were then denoised using HFC, regardless of whether feedback had been applied (and hence real-time HFC had been applied). This was done to both feedback on and off conditions so that the only difference between them was the real-time correction, in order to fairly compare them, and to help reduce noise in the feedback on case which the real-time feedback failed to correct. We applied three band-stop filters at 50 Hz, 120 Hz and 83 Hz corresponding respectively to line noise, the OptiTrack camera sampling frequency, and the mixing frequency of the 840 Hz harmonic of the OptiTrack sampling and the 923 Hz OPM modulation frequency. We then applied a high-pass filter at 2 Hz and a low-pass filter at 40 Hz. All of these filters were 5th order Butterworth filters applied bidirectionally to achieve zero-phase shift. The data were then epoched into 700 ms trials around the tones (200 ms pre-stimulus, 500 ms post-stimulus) and the blocks combined based on whether feedback was on or off and whether the participant was seated or walking (i.e. as though there were four approximately 10-minute recordings for each participant, one for each condition). The blocks were combined to increase the statistical power of the experiment. This left four datasets for each participant with at least 1122 trials in each.

To allow fair comparison between the feedback off and on conditions, we randomly selected 1120 trials from each dataset for analysis. However, after rejecting any trials containing saturated data from these 1120 trials, there were considerably fewer trials in the feedback off - walking case. This is the major advantage of the feedback - that data can be recorded when previously the background field was too high - and so in the walking condition, we performed the auditory evoked analysis with 457, 531 and 638 trials in the feedback off case and 1087, 1120 and 1118 trials when feedback was used for participant 1′s 1st and 2nd recordings and participant 2 respectively. The full list of the number of trials in each condition is given in Table 1.

OP-MEG data were averaged and a one sample *t*-test performed across trials. Sensor level field-maps were produced for the evoked response between 95 ms and 105 ms of only the radially orientated channels. We then projected this data to the source space using all channels, both those radial to the scalp and those tangential to it.

The forward model for each participant was created from their MRI. A single shell model was used based on the inner skull surface (Nolte, 2003). Minimum norm inversion was used (Hämäläinen and Ilmoniemi, 1994) for source inversion, as implemented in SPM12 (Litvak et al., 2011; López et al., 2014). The source space was the cortical surface, constructed by warping the SPM template mesh based on the participant's MRI, with 5124 sources on the cortical surface with their orientation fixed perpendicular to it. For each source on the mesh, we performed one-sample t-tests over trials. These t-statistic images were then spatially smoothed in SPM with an 8 mm (full width half maximum) kernel. Additionally, the source time series at the auditory cortices was estimated by a weighted sum of the OPM recordings based on the lead fields at the cortical mesh vertices closest to each auditory cortex. Where $X\in R^{2\times n}$ is the source-level time series, $X=L^{†}Y$ where n is the number of timepoints, $L\in R^{2\times n}$ is the lead fields (from the single shell model in SPM) from the left and right auditory cortices and Y is the averaged OPM data.

## 3 Results

### 3.1 Environmental noise recording

**Fig. 3:**
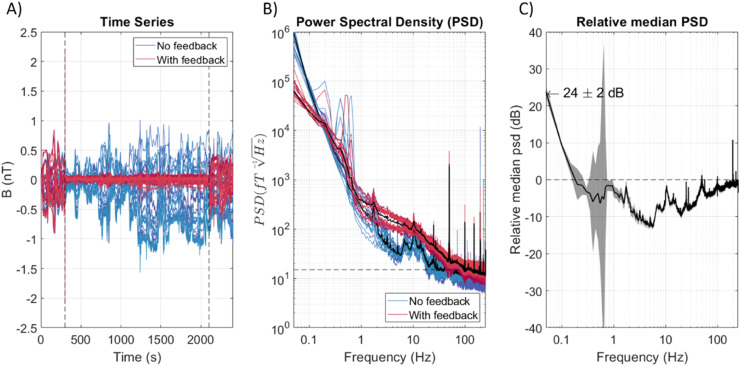
The time series (A), power spectral density (PSD) (B) and corresponding relative PSD (C) for a 40-minute recording in which feedback was turned on between times 5 min and 35 min for 22 channels, with no feedback on 20 channels. The pink lines are sensors for which feedback was used, the blue lines are sensors where feedback was not used. In the PSD, the median (over channels) value for each case is shown as a black line. The relative median PSD shows the difference between these median curves in decibels. A value above 0 would imply that the feedback was beneficial, while below zero it is detrimental. The range (the shaded grey area) is calculated from the standard error of the median (over channels) of each set of feedback or without feedback channels.

Fig. 3A shows recordings from 42 stationary OPM channels in an empty room, where feedback was turned on for 22 channels (shown in pink/red). The channels were recorded simultaneously. Feedback was turned on after 5 minutes of recording, after which there is a clear decrease in the magnitude of the OPM recordings where feedback was included. The power spectral density (PSD) of each channel is shown in Fig. 3B. The median PSD (over channels) for the feedback off and on groups is shown as the two thick, black lines. The PSD appears lower, implying a decrease in noise, when feedback is on for frequencies below 0.2 Hz but higher for frequencies above 1 Hz. This is reiterated in Fig. 3C, where the median shielding factor is shown, with the range given by the standard deviation of the feedback on and off groups, propagated through to the relative difference. As the feedback on and off cases were recorded simultaneously, it is not possible to compare a channel with itself. Therefore, there may be inaccuracies due to the differing noise profiles and locations of the different channels, which likely explains the high standard deviation at 0.8 Hz, but this is expected to average out when considering all of the on or off channels as a group. This relative PSD implies that the feedback is beneficial at low frequencies, with the maximum at 0.05 Hz (the lowest frequency possible to interpret as the PSD was calculated from 20 s chunks of data) of 24±2 dB. However, above approximately 0.5 Hz, the feedback is detrimental and raises the noise floor of the sensors.

In the recordings in Fig. 3, a 1 Hz low-pass filter was applied to the output of the feedback model. The comparison with the unfiltered case is shown in Supplementary Figure 2. It appears that the filter reduces the performance of the feedback at low frequencies and perhaps reduces the frequency at which the feedback becomes detrimental, but it does improve the feedback performance at frequencies above 5 Hz. We consider the source of the increase in noise above 1 Hz from introducing feedback in greater detail in the supplementary material, and show that much of this additional noise can be recreated by considering the timing of the feedback.

### 3.2 Auditory paradigm

**Fig. 4:**
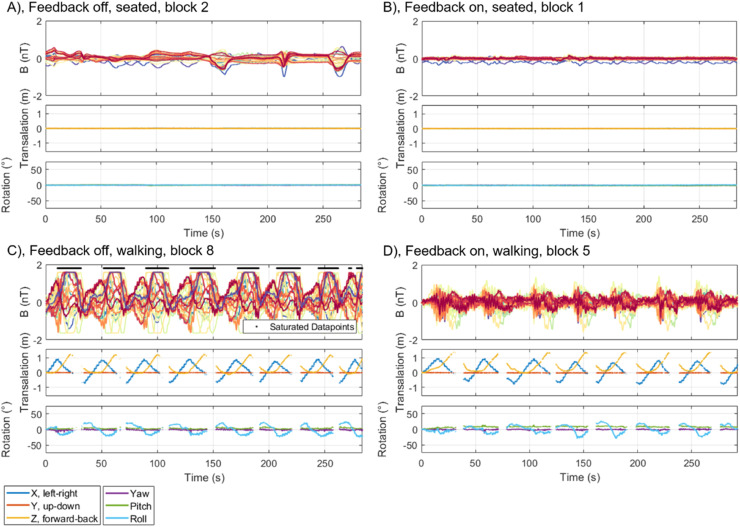
Full, raw time series for an example block of each condition in the auditory experiment. The OPM recordings are shown on top with the participant's position and rotation, as recorded with the OptiTrack motion tracking system, shown below. All OPM channels are shown, each in a different colour. The black crosses mark datapoints that have been marked as one or more OPMs having saturated. The gaps in the OptiTrack recordings indicate that more than 3 markers were occluded. The left two panes are the runs with feedback off, the right two have feedback on.

The raw recordings for an example block of each condition are shown for participant 1 in Fig. 4. The recordings for all blocks can be found in the supplementary material. There is clear evidence of the impact of feedback on the time series. Most apparent, when the feedback is off, the sensors frequently saturate. We rejected any auditory trial where at least 1 OPM channel was saturated. This meant rejecting 51.6% of feedback off walking trials and 1.0% of feedback on walking trials, leaving 457 feedback off walking trials for participant 1′s initial recording, 531 feedback off trials for participant 1′s main recording, and 638 feedback off trials for participant 2′s recording. By comparison, each recording had over 1087 feedback on walking trials.

**Fig. 5:**
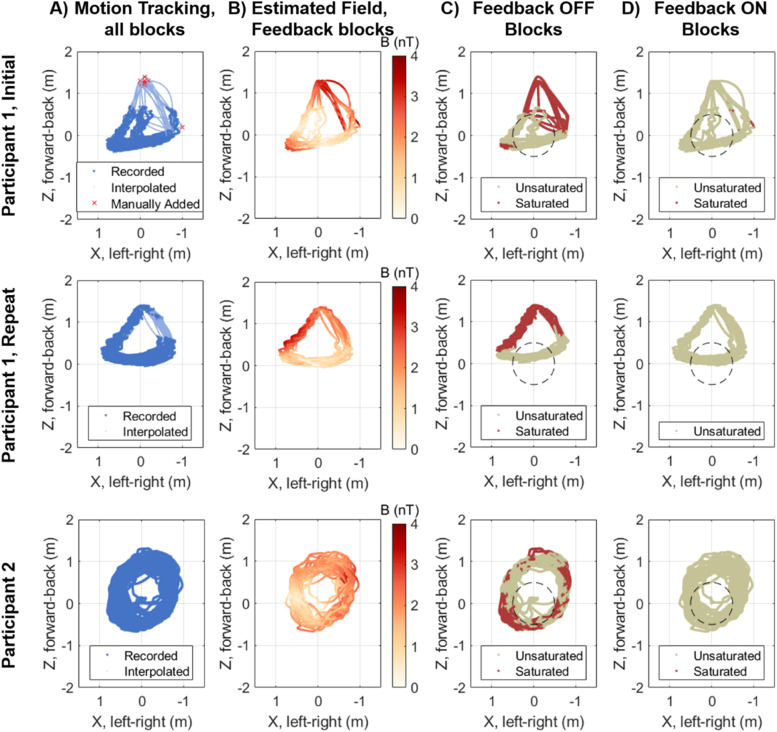
The trajectory of the participant through the room. A) Position as recorded with OptiTrack Flex 13 motion tracking cameras in every run overlaid. Shows the region where motion tracking was successful (dark blue points), where data was manually added (red crosses) and where data was interpolated (light blue points). B) Background magnetic field magnitude over the participant's path in runs with feedback on, as estimated from the intended feedback applied. The field is approximately 3.5 nT at the front and right of the MSR but is below 1 nT in the centre. C) and D) Positions where at least 1 OPM channel was saturated (red) when feedback is off (C) and when feedback is on (D). A black, dashed circle marks a 50 cm radius from the centre of the room.

The majority of these rejected trials fell outside of a 50 cm radius cylinder at the centre of the room, as can be seen from Fig. 5C and Fig. 5D. The number of auditory trials in each feedback condition, classified by whether they were within 50 cm or not, is shown in Table 1. Seated and walking recordings are included in Table 1. If at any time point in the trial, the centre of the participant's cortical mesh (as estimated from the motion tracking recording) was outside of 50 cm of the centre of the room, the trial was counted as outside of 50 cm. To account for the missing motion tracking data, if the gap in the data was less than 0.2 s (and as such linear interpolation had been used to fill the gap), the interpolated data was assumed to be true. For larger gaps, it was assumed that the participant was outside of this 50 cm radius cylinder. As can be seen from Table 1, most trials within 50 cm of the centre of the room did not contain a single saturated datapoint. However, outside of 50 cm, 61% of trials were saturated when feedback was not used. When feedback was introduced, this fell to 1.3%, more than doubling the number of unsaturated trials in this region.

**Table 1:** Number of trials across all recordings (participant 1 initial, participant 1, participant 2, seated and walking). Formatted as unsaturated/total. Unsaturated is any trial where no sensor channel is saturated at any point during the trial. Most of the trials within the central 50 cm are unsaturated (99.5%) but for those outside, only 38.8% of feedback off trials are unsaturated, compared with 98.7% of feedback on trials.

	Number of Trials (unsaturated/total)		
	Feedback off	Feedback on	TOTAL
Within 50 cm	2785/2811 (99.1%)	2932/2932 (100%)	5717/5743 (99.5%)
Outside 50 cm	1081/2789 (38.8%)	2633/2668 (98.7%)	3714/5457 (68.1%)
TOTAL	3866/5600 (69.0%)	5565/5600 (99.4%)	

The position of the participants within the MSR for all twelve walking blocks is shown in Fig. 5A. The edges of the figure indicate the walls of the MSR. The participant was constrained to the front portion of the room (Z>−0.5m) due to the limited length of the cables for the OPMs and auditory stimulus earphones. The very front of the room (high Z in Fig. 5) was particularly badly sampled by the motion tracking system, as it is normally not used for OP-MEG recordings due to the relatively high background magnetic field (∼4nT). The positions which were estimated from a simultaneously recorded video during participant 1′s initial recording are shown by the red crosses in Fig. 5A. The smaller, lighter coloured points in Fig. 5A were interpolated from the motion tracking recordings. The range of movement (with and without the estimated positions) is given in Supplementary Table 1. These suggest that the degree of movement is comparable between blocks within recording session.

For feedback on walking blocks, the background magnetic field was estimated from the feedback which was applied at each position. The estimated field magnitude is shown in Fig. 5B. This was not possible for blocks without feedback, due to the large number of saturated (and consequently unrepresentative of the magnetic field) datapoints, as shown in Fig. 5C and Fig. 4. The magnetic field is estimated to be approximately 3.5 nT at the front of the MSR, while it is below 2 nT within a 50 cm radius of the centre of the room at the participants' height (which puts these recordings at approximately 70 cm above the true centre of the room). The increased background magnetic field meant that sensors were more frequently observed to saturate outside of this 50 cm radius, as shown in Fig. 5C. However, the area over which the OPMs saturated was considerably reduced when feedback was introduced, as shown in Fig. 5D.

**Fig. 6:**
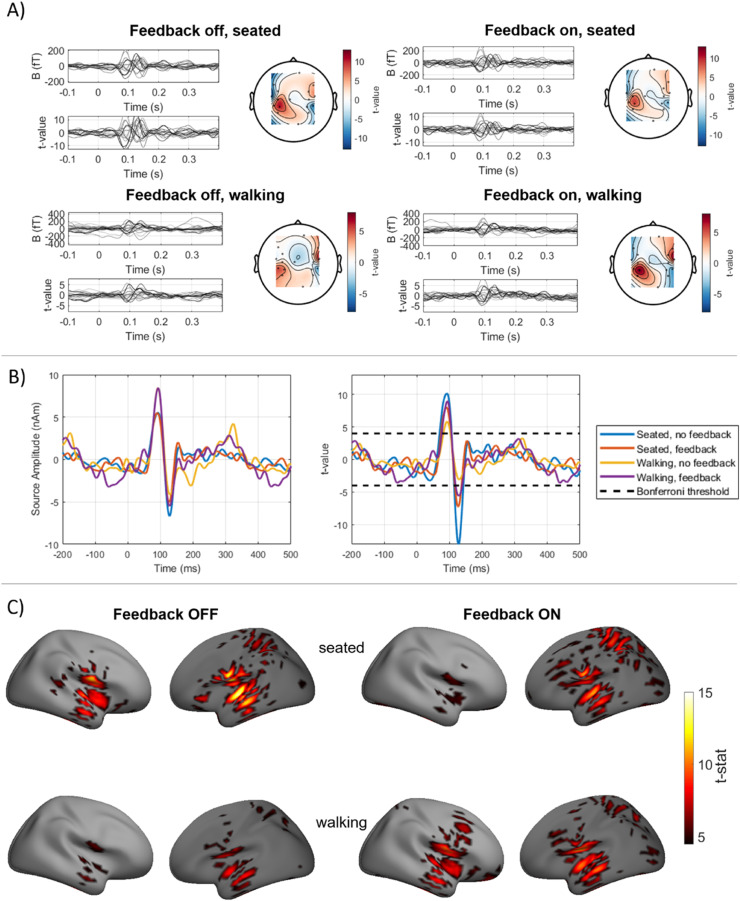
Auditory evoked responses from participant 1. A) Sensor level auditory evoked response. Left, butterfly plot of all sensors with colour scaled by distance from auditory cortices (black closest, light grey furthest). Right, topography of response between 95 ms and 105 ms only for radially orientated (Y) sensor channels. Left, feedback off; right, feedback on. Top, seated; bottom, walking. B) Reconstructed evoked response waveforms at the left auditory cortex for each condition. The significance threshold after Bonferroni correction for multiple comparisons is indicated with a dashed black line. C) T-statistic source maps at 100 ms when feedback is off (left) and on (right). Top; seated condition, bottom; walking.

**Fig. 7:**
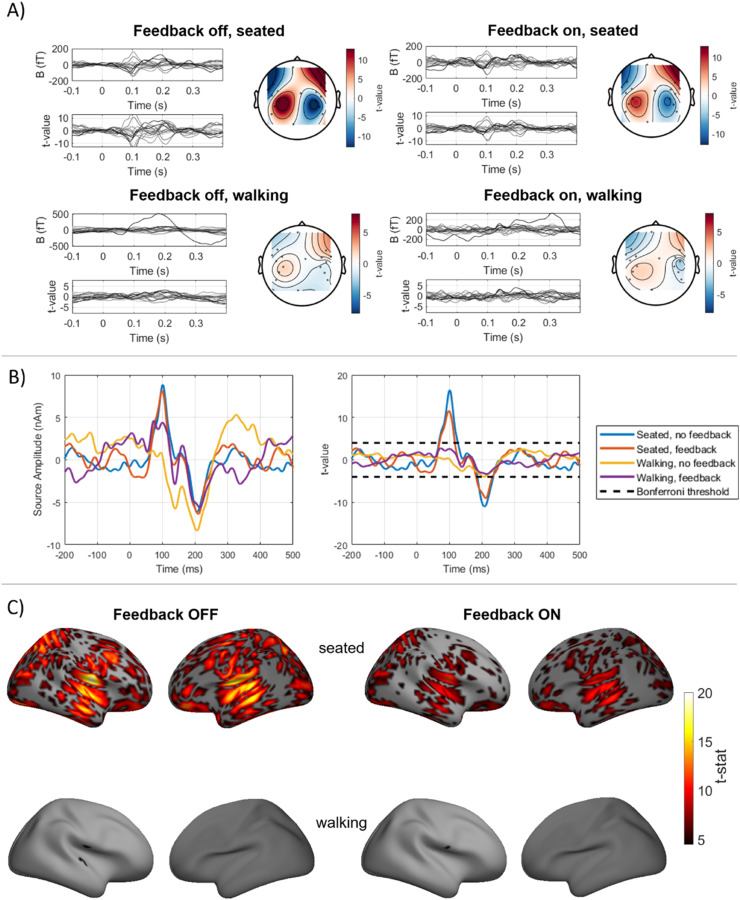
Auditory evoked response for participant 2. A) Sensor level auditory evoked response. Left, butterfly plot of all sensors with colour scaled by distance from auditory cortices (black closest, light grey furthest). Right, topography of response between 80 ms and 120 ms only for radial (Y) sensor channels. Left, feedback off; right, feedback on. Top, seated; bottom, walking. B) Reconstructed evoked response waveforms at the left auditory cortex for each condition. The significance threshold after Bonferroni correction for multiple comparisons is indicated with a dashed black line. C) T-statistic source maps at 100 ms when feedback is off (left) and on (right).

Looking at only the un-saturated trials, the auditory evoked responses for participant 1′s session 2 recording are shown in Fig. 6, alongside participant 2′s recording in Fig. 7. The results for participant 1′s initial recording can be found in the supplementary material. The sensor level auditory responses are shown in Fig. 6A and Fig. 7A. In all cases (feedback off and on, seated and walking), there is evidence for the M100 auditory evoked response with bilateral sources. However, there is a decrease in the t-statistic at 100 ms when feedback is introduced when seated. On average between both participants, using the square of the maximum t-statistic between 95 ms and 105 ms as a measure of the SNR (i.e. a power ratio), when feedback is introduced when seated, there is a 46.35% decrease in SNR. However, when feedback is introduced when walking, due to the larger number of trials, there is an average increase in SNR (at the sensor level) of 17.06%.

The source-level time series at the vertex of the cortical mesh closest to the left auditory cortex is shown for each condition in Fig. 6B and Fig. 7B. The significance threshold (*p* < 0.05, after Bonferroni correction) is also shown. The evoked waveforms appear similar in morphology whether feedback was used or not. This suggests that the feedback has not considerably distorted the auditory signal of interest. As was seen at the sensor level, the statistical significance of the feedback-on condition is lower than the feedback-off condition when seated, but is higher when walking. In the seated case, this is likely due to the increase in noise from the feedback, as shown in Fig. 3, while when walking, this is likely due to the larger number of trials in the feedback on case. There were, for participant 1, 2.11 times as many trials in the feedback on case as feedback off and so the SNR would be expected to increase by the same amount. In the seated condition, on average over both participants, the SNR in the left auditory cortex decreased by 47.18% when feedback was introduced and 40.09% in the right auditory cortex. In the walking condition, the SNR increased by 144±2% in both auditory cortices for participant 1, while the small amplitude of the responses from participant 2 gave inconsistent results, and led to an increase of 4175% in the left auditory cortex and a decrease of 8% in the right auditory cortex.

The auditory evoked response between 0 ms and 200 ms was estimated in SPM and using minimum norm estimation (MNE). We then estimated the time-series at each vertex of the cortical mesh, using the weights from the minimum norm reconstruction. The statistical maps of the 100 ms responses for are shown in Fig. 6C and Fig. 7C. The maps have been thresholded at the *p* < 0.05 level corrected for multiple comparisons with Bonferroni correction. In all conditions, the 100 ms response appears to originate near to the auditory cortices. For participant 1, based on the AAL atlas (Tzourio-Mazoyer et al., 2002) and interpolated with Field Trip to the SPM cortical mesh, the maximum t-value was in the left superior temporal lobe for both seated conditions and in the left Rolandic operculum, adjacent to the primary auditory cortex in the walking conditions. For participant 2, the maximum t-value was in the right Rolandic operculum in all conditions. The feedback on walking case has higher statistical power than the feedback off walking condition (in line with the larger number of trials and consistent with Fig. 6B and Fig. 7B) and, for participant 1, more supra-threshold vertices. This result is stronger for participant 1 than participant 2, perhaps due to the higher degree of movement, rotation and speed of participant 2 when walking (seen in Fig. 5 and Supplementary Table 1).

## 4 Discussion

We have demonstrated a model-based method for feedback correction with an array of magnetometers, which reduced the very slow drifts in the background magnetic field by a factor of approximately 24 dB. We demonstrated that introducing this feedback to OP-MEG measurements during movements of up to 2 m reduced the changes in background field due to movement and so increased the number of analysable trials by keeping the OPMs within their dynamic range.

This is the first demonstration of a field nulling system using the on-sensor coils during an OP-MEG experiment and large translational movements. Existing closed-loop OPMs generally employ a proportional or proportional-integral-derivative (PID) control directly on the OPM output (Lee et al., 2014; Nardelli et al., 2020), or rely on reference sensors fixed to the main sensor array (Robinson et al., 2022). While PID controllers on the OPM output have been successful in minimising changes in background magnetic fields at SERF OPMs in real-time, introducing a model step has some theoretical advantages. Without this modelling, a separate PID would be required for each OPM channel, while the method proposed here requires only a single matrix multiplication with no tuneable parameters. By using the HFC model to estimate the homogeneous field components at any time point, we need only use a proportional control to minimise these estimates, reducing the problem to three terms. Although for practical reasons (see below) our implementation is band-limited, it could theoretically be applied sample by sample and could therefore handle environmental noise or movement noise from across the frequency spectrum.

Importantly, modelling the field in this way allows an estimation of the magnetic field component on an unmeasured OPM axis. This is pertinent for OPMs where the fields on only two axes are recorded, but where interference along the third axis leads to CAPE (Borna et al., 2022), altering the orientation, gain and phase of the recording axes. This is not possible with a proportional-integral-derivative (PID) control based system without implementing a similar model. These CAPE errors are the motivation behind dynamic field compensation (DFC) (Robinson et al., 2022). By implementing real-time HFC, the same goal of controlling the field on the third/unmeasured OPM axis could be achieved without the need for additional hardware.

In our experiments, as we did not control the field on the unmeasured axis, regardless of whether real time correction was included, the recorded data will have been influenced by CAPE (Borna et al., 2022). This is likely partially responsible for the smaller auditory evoked responses when the participant was walking. It will also have reduced the performance of real-time correction as the HFC model will have been limited by the calibration error on the OPM recordings (Tierney et al., 2022).

This method was detrimental at frequencies above 2 Hz, raising the noise floor by a factor of approximately 10 dB below 50 Hz. Due to this noise penalty, this real-time correction is perhaps best considered currently as an option only when large subject movement or other external noise is expected. We were able to reduce but not remove the issue by applying a filter to the HFC model output. The main issue here is the finite amount of time it takes to acquire, process and feedback the predicted field. In this implementation, this time-lag is approximately 41 ms (Supplementary Figure 6). Consequently, the predictions always lag the measurements and this (in addition to the quantized feedback every 10 ms) introduces noise. While the fundamental limit on the speed of the feedback is in the OPM hardware and is discussed in the following paragraph, there is also scope to improve the coding.

In two key regards, our system is limited by the OPM hardware, which was not designed for this purpose. Firstly, the digital-to-analogue converters (DACs) used to produce the requested magnetic fields are 16-bit. The least significant bit (LSB - smallest possible field change) of each coil is approximately 3.1 pT or 1.8 pT for the Z and Y axes respectively (although this does depend on the OPM). This leads to quantisation noise ($\sqrt{LSB^{2}/12}$) of 0.895 pT_rms_ and 0.520 pT_rms_ on each axis respectively. Additionally, the command to produce a particular field is sent via serial connection from the computer to the OPMs. This is a slow connection inevitably leading to a delay between intending to send a value and it being applied to the OPMs. This could be alleviated by future changes to the OPM control electronics. There was also some jitter on the timing of the fields sent to the OPM channels. In a separate experiment, in which we sent a pulse to the OPM channels every 1 s, we found this to usually be less than 3 ms. However, when a command was sent to the OPMs before the previous one had been fully processed, there was an additional 15 ms delay. In the 300 pulses we tested, this occurred 19% of the time.

Additionally, we have not fully considered any forward modelling errors introduced by applying HFC to the recordings in real-time. As is shown by Tierney et al. (2021), by multiplying the data by a projection matrix $MN^{†}$, it is necessary to multiply the lead fields of the forward model by the same matrix. This correction was applied when HFC was used in pre-processing the data. However, due to the feedback being applied every 10 ms to each axis (and the correction to the Y-axis preceding the Z-axis by 5 ms), the real-time correction made cannot be so easily summarised as a projection matrix. The lack of this lead field correction also makes the results in Fig. 6C difficult to interpret, although some confidence can be gained from the temporal properties of the signal and the correction: the real-time correction was low-pass filtered at 1 Hz while the signal was high-pass filtered at 2 Hz.

We have tested this system with two compliant, healthy adult participants performing large, natural but reasonably smooth movements. A considerable opportunity for such a feedback system would be to record OP-MEG from population groups such as children, where movements would likely be exaggerated and perhaps more sudden. Paediatric OP-MEG has been recorded while seated (Boto et al., 2022; Feys et al., 2022; Hill et al., 2019), but larger movements are yet to be recorded. There is also potential for use with clinical populations where movements, such as tremors or seizures, may be sudden. In our system, the maximum update speed for a given axis is 10 ms and the feedback cannot correct for interference above 4 Hz (see supplementary material). This limits the feedback performance, although fortunately most movements are below 4 Hz. Timing of the feedback is likely to be critical to improving the performance for fast and sudden movements.

We were fortunate to have person-specific scanner-casts to hold the OPMs, from which we were able to determine the OPM positions and channel orientations. We would expect the feedback system to work equally well with any rigid scanner-cast with a similar sensor coverage. One of the advantages of HFC to determine the feedback required is that it does not require any knowledge of the underlying anatomy and so it is only necessary to know the sensor positions and orientations relative to one another. Flexible scanner-casts may provide more of a challenge, as the sensor positions and orientations will change relative to one another over the course of a recording, reducing the accuracy of the HFC model. However, we would hope that any such changes would be small (as they would otherwise limit the source localisation of the OP-MEG), and so while this remains to be tested, real-time feedback as we have implemented it would likely still be beneficial with a flexible sensor array.

If appropriate training data were available, signal space projection (SSP) (Uusitalo and Ilmoniemi, 1997) could alternatively be used to determine the feedback required to the OPMs. Both HFC and SSP involve a projection of the data into a space orthogonal to the background interference. However, in HFC the projection matrix is determined by the geometry of the sensor array, while in SSP, it is determined by the statistical properties of a set of training data. This would be challenging for moving OP-MEG since the background magnetic field changes with space and so the projection matrix would ideally change over time. This means that SSP as it is typically performed, using an empty room recording as a training dataset, would not be appropriate.

A major consideration for feedback applied to the OPM on-board coils is cross-talk, both between sensors and within a single sensor. When a field is applied to a single sensor using the on-board coils, it will impact neighbouring sensors. Additionally, the field produced by the on-board coils is not perfectly homogeneous and is not perfectly orientated along one axis, so correcting the background field on one axis will also alter the field along other axes within the same OPM. We have not considered this in this methodology. Based on (Boto et al., 2018), we estimate that approximately 3% of feedback to a channel will be picked up by a neighbouring OPM. Cross-talk could be mitigated by computing or measuring the cross-talk matrix of an OPM array a-priori and accounting for it in the model of the feedback. Alternatively, there are OPMs with internal coils specifically designed to minimise cross-talk (Nardelli et al., 2019).

Triaxial OPMs promise to have a significant impact on OP-MEG and its applications (Boto et al., 2022). Here we have focussed on dual-axis OPMs, as there is a clear use case for a model-based feedback method to minimise the magnetic field on both the measured and unmeasured axes. Moving to triaxial sensors could, however, be of some benefit for the spherical harmonic model used here, since the additional information would lead to a more accurate model, and would increase the orthogonality between the interference model and the neural signal (Tierney et al., 2022).

Here we have only tested applying the simplest form of HFC in real-time. In this case, the background field is assumed to be locally spatially homogeneous. HFC can, however, be simply extended to incorporate higher order spatial magnetic field terms, including spatial field gradients and curvature terms (Tierney et al., 2022). This is likely to be necessary in environments with higher or more spatially complex magnetic fields. For example, in a magnetic field gradient of 20 nT/m, two OPMs placed 15 cm apart would experience a field difference of 3 nT, and so field gradients would be required in the field model to keep the OPMs below 1.5 nT. Computationally, there is no disadvantage to adding higher order spherical harmonics to the model as the size of the projection matrix $MN^{†}$ is the number of channels to feedback to by the number of channels the model is created from. The only disadvantage is the potential to regress out brain signal and so the number of sensors and their positions and orientations should be considered when deciding on the order of the model. As OP-MEG sensor arrays increase in size, this would be a natural extension to this work.

Increasingly, the research field is moving towards unshielded OP-MEG (Limes et al., 2020; Zhang et al., 2020). In such an environment, background field drifts are likely to be of the order of 100s of nano Tesla, rather than 100s of pico Tesla, assuming that the worst of the static field (of the order of micro Tesla) can be handled by light external passive or active magnetic shielding. To allow OP-MEG recordings in an urban environment without an MSR, on-board feedback such as this is likely to be necessary to minimise these drifts, regardless of the design of the OPM. Therefore, while this system no doubt has limitations, it has considerable promise to allow us to ask questions in a naturalistic environment which previously could not be considered with MEG.

## CRediT authorship contribution statement

**Stephanie Mellor:** Conceptualization, Methodology, Software, Formal analysis, Investigation, Writing – original draft. **Tim M. Tierney:** Conceptualization, Methodology, Software, Resources, Writing – review & editing. **Robert A. Seymour:** Software, Resources, Writing – review & editing. **Ryan C. Timms:** Software, Investigation, Writing – review & editing. **George C. O'Neill:** Software, Resources, Writing – review & editing. **Nicholas Alexander:** Software, Investigation, Resources, Writing – review & editing. **Meaghan E. Spedden:** Resources, Writing – review & editing. **Heather Payne:** Resources, Writing – review & editing. **Gareth R. Barnes:** Conceptualization, Methodology, Software, Resources, Writing – review & editing, Supervision, Funding acquisition.

## Declaration of Competing Interest

This work was partly funded by a Wellcome award which involves a collaboration agreement with QuSpin.

## Data Availability

Analysis code is available on GitHub (https://github.com/stephaniemellor/OPMEGfeedback). The anonymised data that support the findings in this study is available from Zenodo (10.5281/zenodo.7872660).

## Appendix Supplementary materials

Image, application 1

## References

[bib0001] Altarev I., Fierlinger P., Lins T., Marino M.G., Nießen B., Petzoldt G., Reisner M., Stuiber S., Sturm M., Taggart Singh J., Taubenheim B., Rohrer H.K., Schläpfer U. (2015). Minimizing magnetic fields for precision experiments. J. Appl. Phys..

[bib0002] Baillet S. (2017). Magnetoencephalography for brain electrophysiology and imaging. Nat. Neurosci..

[bib0003] Borna A., Iivanainen J., Carter T.R., McKay J., Taulu S., Stephen J., Schwindt P.D.D. (2022). Cross-Axis projection error in optically pumped magnetometers and its implication for magnetoencephalography systems. Neuroimage.

[bib0004] Boto E., Holmes N., Leggett J., Roberts G., Shah V., Meyer S.S., Muñoz L.D., Mullinger K.J., Tierney T.M., Bestmann S., Barnes G.R., Bowtell R., Brookes M.J. (2018). Moving magnetoencephalography towards real-world applications with a wearable system. Nature.

[bib0005] Boto E., Shah V., Hill R.M., Rhodes N., Osborne J., Doyle C., Holmes N., Rea M., Leggett J., Bowtell R., Brookes M.J. (2022). Triaxial detection of the neuromagnetic field using optically-pumped magnetometry: feasibility and application in children. Neuroimage.

[bib0006] Chowdhury, J., 2020. Stable Structures For Nonlinear Biquad Filters, in: Proceedings of the 23rd International Conference on Digital Audio Effects (DAFx-2020). Vienna, Austria, p. 7.

[bib0007] Feys O., Corvilain P., Aeby A., Sculier C., Holmes N., Brookes M., Goldman S., Wens V., De Tiège X. (2022). On-scalp optically pumped magnetometers versus cryogenic magnetoencephalography for diagnostic evaluation of epilepsy in school-aged children. Radiology.

[bib0008] Garrido M.I., Friston K.J., Kiebel S.J., Stephan K.E., Baldeweg T., Kilner J.M. (2008). The functional anatomy of the MMN: a DCM study of the roving paradigm. Neuroimage.

[bib0009] Hämäläinen M., Hari R., Ilmoniemi R.J., Knuutila J., Lounasmaa O.V. (1993). Magnetoencephalography theory, instrumentation, and applications to noninvasive studies of the working human brain. Rev. Mod. Phys..

[bib0010] Hämäläinen M.S., Ilmoniemi R.J. (1994). Interpreting magnetic fields of the brain: minimum norm estimates. Med. Biol. Eng. Comput..

[bib0011] Happer W., Tang H. (1973). Spin-exchange shift and narrowing of magnetic resonance lines in optically pumped alkali vapors. Phys. Rev. Lett..

[bib0012] Hill R.M., Boto E., Holmes N., Hartley C., Seedat Z.A., Leggett J., Roberts G., Shah V., Tierney T.M., Woolrich M.W., Stagg C.J., Barnes G.R., Bowtell R.R., Slater R., Brookes M.J. (2019). A tool for functional brain imaging with lifespan compliance. Nat. Commun..

[bib0013] Hill R.M., Devasagayam J., Holmes N., Boto E., Shah V., Osborne J., Safar K., Worcester F., Mariani C., Dawson E., Woolger D., Bowtell R., Taylor M.J., Brookes M.J. (2022). Using OPM-MEG in contrasting magnetic environments. Neuroimage.

[bib0014] Holmes N., Leggett J., Boto E., Roberts G., Hill R.M., Tierney T.M., Shah V., Barnes G.R., Brookes M.J., Bowtell R. (2018). A bi-planar coil system for nulling background magnetic fields in scalp mounted magnetoencephalography. Neuroimage.

[bib0015] Holmes N., Rea M., Hill R.M., Boto E., Stuart A., Leggett J., Edwards L.J., Rhodes N., Shah V., Osborne J., Fromhold T.M., Glover P., Montague P.R., Brookes M.J., Bowtell R. (2021). Naturalistic hyperscanning with wearable magnetoencephalography. bioRxiv.

[bib0016] Holmes N., Tierney T.M., Leggett J., Boto E., Mellor S., Roberts G., Hill R.M., Shah V., Barnes G.R., Brookes M.J., Bowtell R. (2019). Balanced, bi-planar magnetic field and field gradient coils for field compensation in wearable magnetoencephalography. Sci. Rep..

[bib0017] Iivanainen J., Zetter R., Grön M., Hakkarainen K., Parkkonen L. (2019). On-scalp MEG system utilizing an actively shielded array of optically-pumped magnetometers. Neuroimage.

[bib0018] Johnson C., Schwindt P.D.D., Weisend M. (2010). Magnetoencephalography with a two-color pump-probe, fiber-coupled atomic magnetometer. Appl. Phys. Lett..

[bib0019] Kim K., Begus S., Xia H., Lee S.K., Jazbinsek V., Trontelj Z., Romalis M.V. (2014). Multi-channel atomic magnetometer for magnetoencephalography: a configuration study. Neuroimage.

[bib0020] Lee H.J., Shim J.H., Moon H.S., Kim K. (2014). Flat-response spin-exchange relaxation free atomic magnetometer under negative feedback. Opt. Express.

[bib0021] Limes M.E., Foley E.L., Kornack T.W., Caliga S., McBride S., Braun A., Lee W., Lucivero V.G., Romalis M.V. (2020). Portable magnetometry for detection of biomagnetism in ambient environments. Phys. Rev. Appl..

[bib0022] Litvak V., Mattout J., Kiebel S., Phillips C., Henson R., Kilner J., Barnes G., Oostenveld R., Daunizeau J., Flandin G., Penny W., Friston K. (2011). EEG and MEG data analysis in SPM8. Comput. Intell. Neurosci..

[bib0023] López J.D., Litvak V., Espinosa J.J., Friston K., Barnes G.R. (2014). Algorithmic procedures for Bayesian MEG/EEG source reconstruction in SPM. Neuroimage.

[bib0024] Mellor S.J., Tierney T., O'Neill G., Alexander N., Seymour R., Holmes N., Lopez J.D., Hill R., Boto E., Rea M., Roberts G., Leggett J., Bowtell R., Brookes M.J., Maguire E., Walker M., Barnes G. (2021). Magnetic field mapping and correction for moving OP-MEG. IEEE Trans. Biomed. Eng..

[bib0025] Meyer S.S., Bonaiuto J., Lim M., Rossiter H., Waters S., Bradbury D., Bestmann S., Brookes M., Callaghan M.F., Weiskopf N., Barnes G.R. (2017). Flexible head-casts for high spatial precision MEG. J. Neurosci. Methods.

[bib0026] Nardelli N.V., Krzyzewski S.P., Knappe S.A. (2019). Reducing crosstalk in optically-pumped magnetometer arrays. Phys. Med. Biol..

[bib0027] Nardelli N.V., Perry A.R., Krzyzewski S.P., Knappe S.A. (2020). A conformal array of microfabricated optically-pumped first-order gradiometers for magnetoencephalography. EPJ Quantum Technol..

[bib0028] Nolte G. (2003). The magnetic lead field theorem in the quasi-static approximation and its use for magnetoencephalography forward calculation in realistic volume conductors. Phys. Med. Biol..

[bib0029] Oostenveld R., Fries P., Maris E., Schoffelen J.M. (2011). FieldTrip: open source software for advanced analysis of MEG, EEG, and invasive electrophysiological data. Comput. Intell. Neurosci..

[bib0030] Osborne J., Orton J., Alem O., Shah V. (2018). Steep Dispersion Engineering and Opto-Atomic Precision Metrology XI. Presented at the Steep Dispersion Engineering and Opto-Atomic Precision Metrology XI.

[bib0031] Peirce J. (2009). Generating stimuli for neuroscience using PsychoPy. Front. Neuroinformatics.

[bib0032] Robinson S.E., Andonegui A.B., Holroyd T., Hughes K.J., Alem O., Knappe S., Maydew T., Griesshammer A., Nugent A. (2022). Cross-axis dynamic field compensation of optically pumped magnetometer arrays for MEG. Neuroimage.

[bib0033] Seymour R.A., Alexander N., Mellor S., O'Neill G.C., Tierney T.M., Barnes G.R., Maguire E.A. (2022). Interference suppression techniques for OPM-based MEG: opportunities and challenges. Neuroimage.

[bib0034] Seymour R.A., Alexander N., Mellor S., O'Neill G.C., Tierney T.M., Barnes G.R., Maguire E.A. (2021). Using OPMs to measure neural activity in standing, mobile participants. Neuroimage.

[bib0035] Shah V., Knappe S., Schwindt P.D.D., Kitching J. (2007). Subpicotesla atomic magnetometry with a microfabricated vapour cell. Nat. Photonics.

[bib0036] Shah V.K., Wakai R.T. (2013). A compact, high performance atomic magnetometer for biomedical applications. Phys. Med. Biol..

[bib0037] Smith J.O. (2007).

[bib0038] Tierney T.M., Alexander N., Mellor S., Holmes N., Seymour R., O'Neill G.C., Maguire E.A., Barnes G.R. (2021). Modelling optically pumped magnetometer interference in MEG as a spatially homogeneous magnetic field. Neuroimage.

[bib0039] Tierney T.M., Holmes N., Mellor S., López J.D., Roberts G., Hill R.M., Boto E., Leggett J., Shah V., Brookes M.J., Bowtell R., Barnes G.R. (2019). Optically pumped magnetometers: from quantum origins to multi-channel magnetoencephalography. Neuroimage.

[bib0040] Tierney T.M., Mellor S., O'Neill G.C., Timms R.C., Barnes G.R. (2022). Spherical harmonic based noise rejection and neuronal sampling with multi-axis OPMs. Neuroimage.

[bib0041] Tzourio-Mazoyer N., Landeau B., Papathanassiou D., Crivello F., Etard O., Delcroix N., Mazoyer B., Joliot M. (2002). Automated anatomical labeling of activations in SPM using a macroscopic anatomical parcellation of the MNI MRI single-subject brain. Neuroimage.

[bib0042] Uusitalo M.A., Ilmoniemi R.J. (1997). Signal-space projection method for separating MEG or EEG into components. Med. Biol. Eng. Comput..

[bib0043] Xia H., Ben-Amar Baranga A., Hoffman D., Romalis M.V. (2006). Magnetoencephalography with an atomic magnetometer. Appl. Phys. Lett..

[bib0044] Zhang R., Xiao W., Ding Y., Feng Y., Peng X., Shen L., Sun C., Wu T., Wu Y., Yang Y., Zheng Z., Zhang X., Chen J., Guo H. (2020). Recording brain activities in unshielded Earth’s field with optically pumped atomic magnetometers. Sci. Adv..

