# Supplementary material for: Real-time, model-based magnetic field correction for moving, wearable MEG
Source: Neuroimage. 2023 Sep;278:120252. doi: 10.1016/j.neuroimage.2023.120252 (PMC11157691; doi:10.1016/j.neuroimage.2023.120252)
Supplement: Supplementary file 1 [file mmc1.docx]

# Environmental Noise Recording Filters


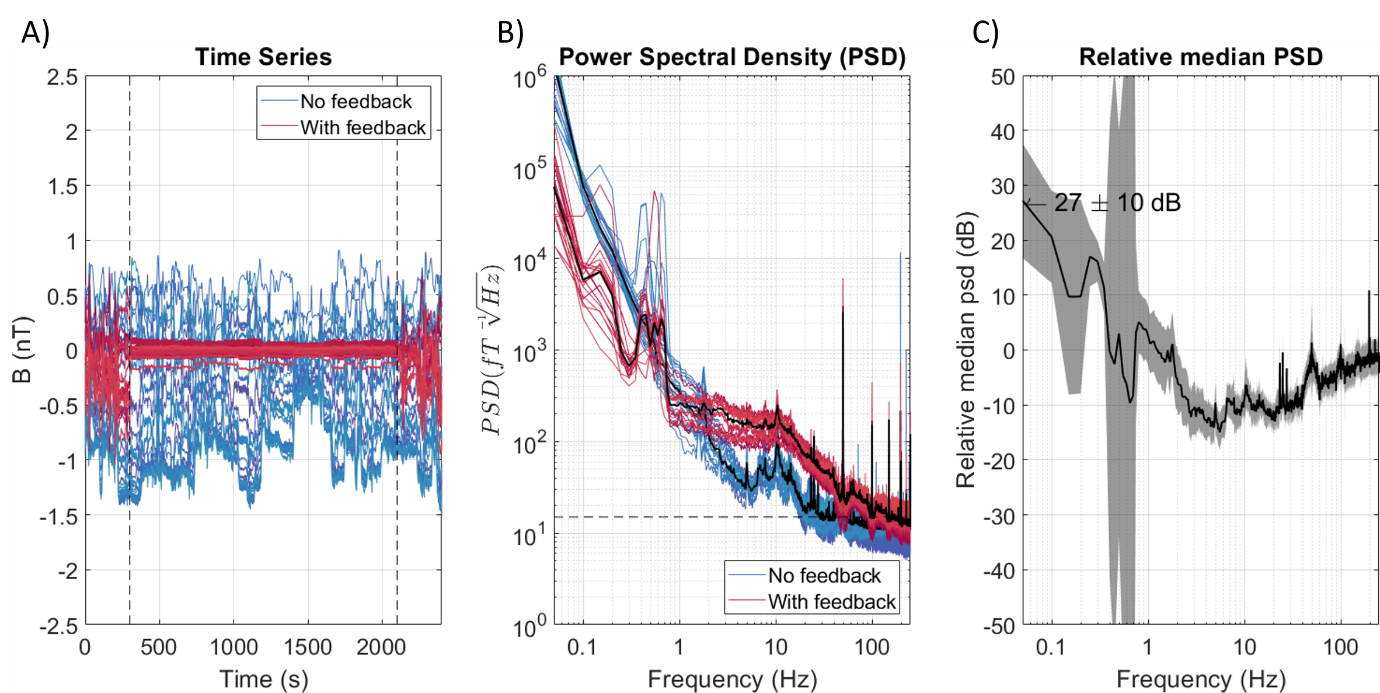


Supplementary Figure 1. Repeat of Figure 3 in the main manuscript but without a low-pass filter on the feedback model output. A) The time series, B) power spectral density (PSD) and C) corresponding relative PSD for a 40-minute recording in which feedback was turned on between times 5 min and 35 min for 22 channels, with no feedback on 20 channels. The pink lines are sensors for which feedback was used, the blue lines are sensors where feedback was not used. In the PSD, the median (over channels) value for each case is shown as a black line. The relative median PSD shows the difference between these median curves in decibels. A value above 0 would imply that the feedback was beneficial, while below zero it is detrimental. The range (the shaded grey area) is calculated from the standard deviation (over channels) of each set of feedback or without feedback channels. The feedback appears to be beneficial at low frequencies but detrimental above 1 Hz.


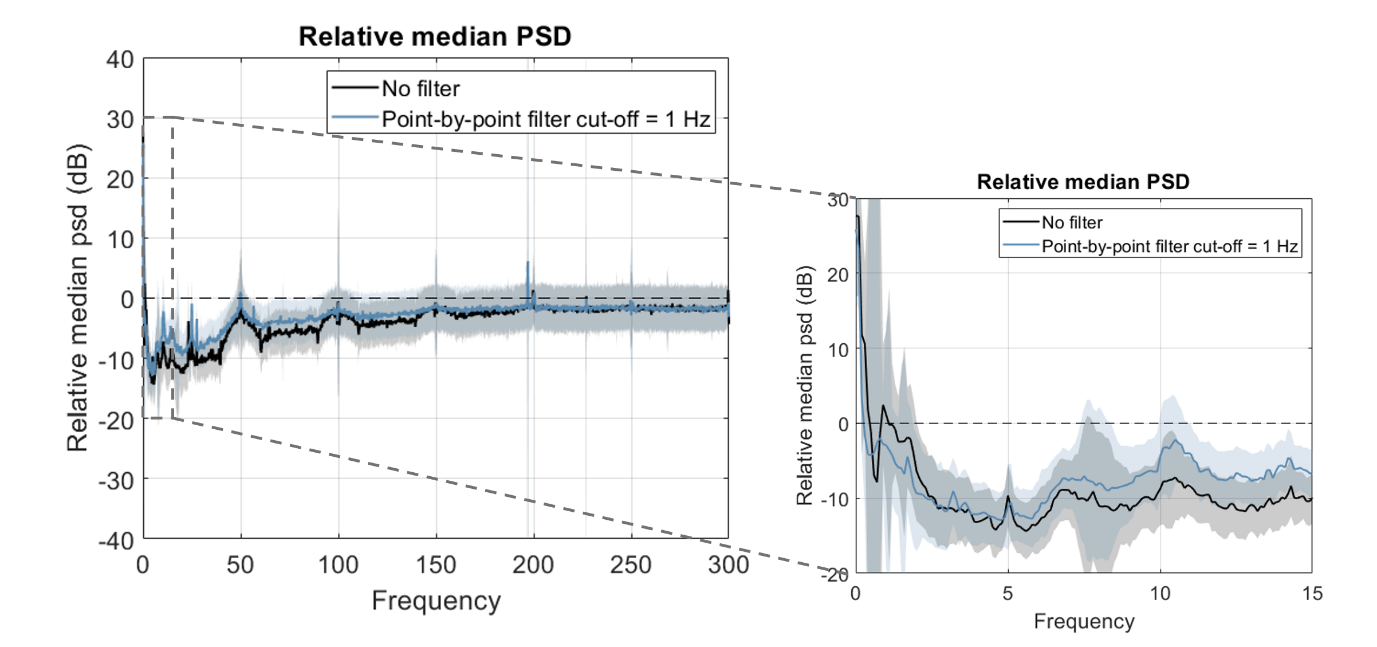


Supplementary Figure 2. Comparison of PSD change when feedback is used without a filter (Supplementary Figure 1) and with a 1 Hz low-pass filter on the model output (Figure 3 of the main manuscript). In each case, feedback was applied to the same 22 OPM channels, while 20 OPM channels recorded background noise for 30 minutes. The introduction of the filter reduces performance below 2 Hz but improves it above 5 Hz.

# Auditory Recordings

## Intended Feedback Power Spectra

The power spectrum of the intended feedback is shown for each feedback block in Supplementary Figure 3. There is a decrease in the intended feedback at and around 100 Hz due to the 10 ms moving average filter. However, the intended feedback broadly follows a 1/f pattern, especially for the walking condition, and so despite the moving average filter, the feedback captures the dominant field components.


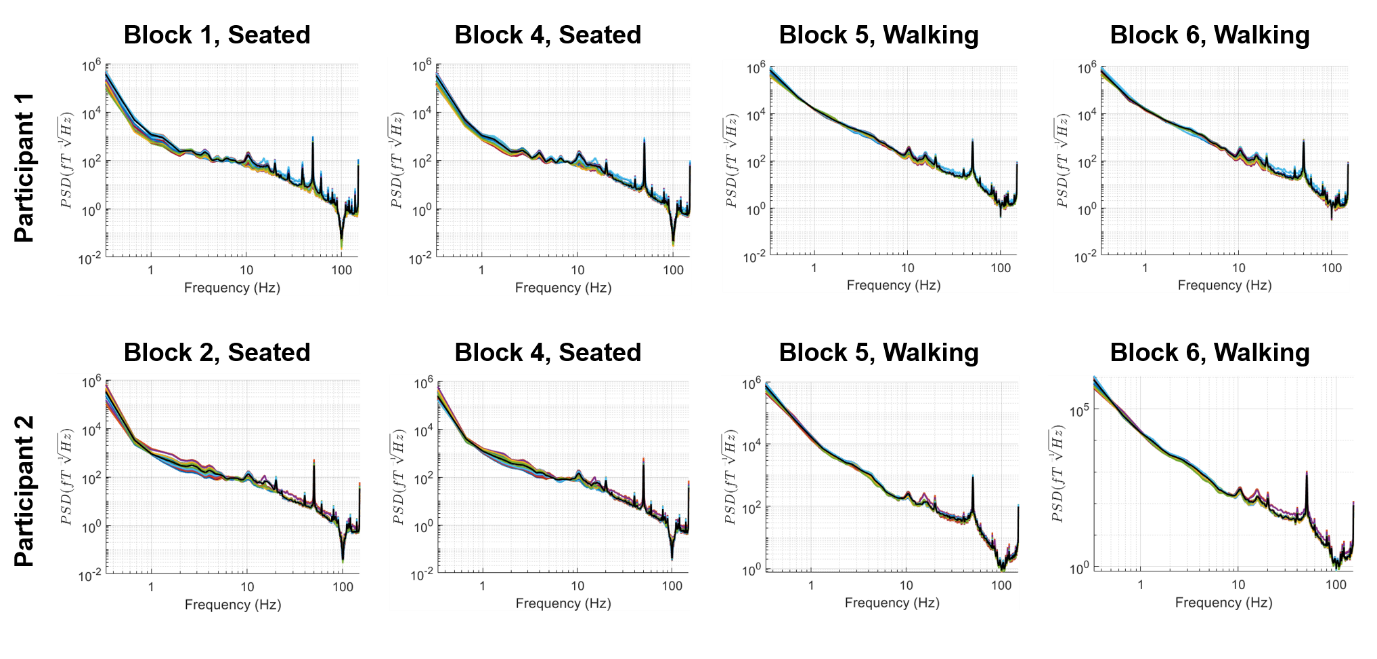


Supplementary Figure 3. The power spectra of the intended feedback for each block where feedback was included for both participants. Each channel is shown in a different colour. The black line shows the median over channels.

## Position Time Courses


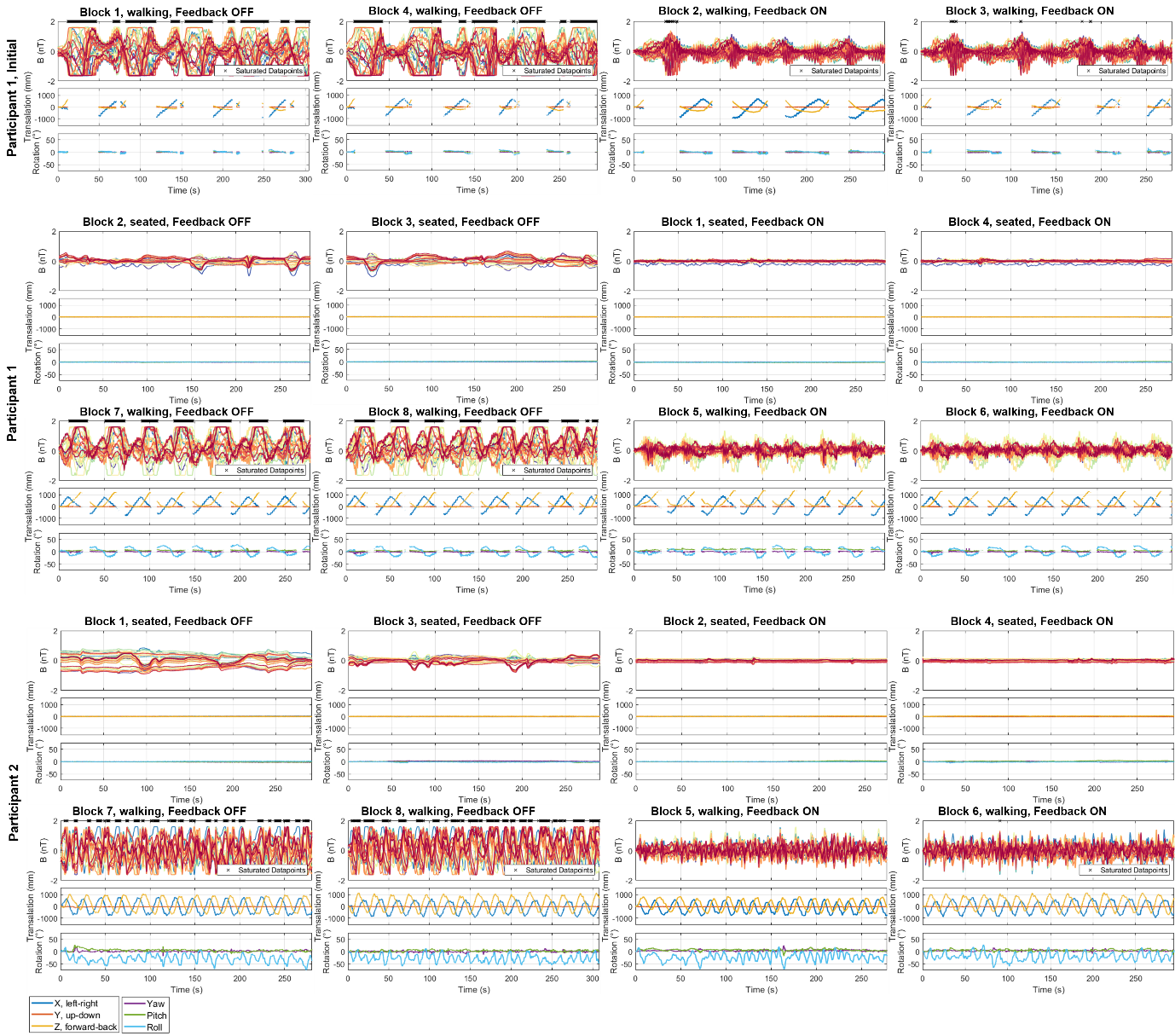


Supplementary Figure 4. The raw data for each block of the auditory experiment before pre-processing (although after downsampling). The OPM recordings are shown on top with the participant’s position and rotation, as recorded with the OptiTrack motion tracking system. All OPM channels are shown, each in a different colour. The black crosses mark datapoints that have been marked as one or more OPMs having saturated. The gaps in the OptiTrack recordings indicate that more than 3 markers were occluded. Repeat of Figure 4 from the main manuscript with all blocks shown.

|  | Block | Range of Position (m) | | | Average speed (m/s) | Range of Rotation (°) | | |
| --- | --- | --- | --- | --- | --- | --- | --- | --- |
|  |  | Left-Right | Up-Down | Forward-Back |  | Yaw | Pitch | Roll |
| Participant 1, Initial | 1 | 1.56 | 0.03 | 0.94 | 0.11 | 21.55 | 13.53 | 3.43 |
|  | 2 | 1.72 | 0.05 | 1.05 | 0.11 | 21.13 | 17.92 | 5.53 |
|  | 3 | 1.57 | 0.10 | 1.07 | 0.09 | 29.38 | 21.98 | 6.96 |
|  | 4 | 1.63 | 0.04 | 1.03 | 0.08 | 22.23 | 11.78 | 5.14 |
| Participant 1 | 5 | 1.76 | 0.04 | 1.44 | 0.16 | 56.85 | 15.48 | 16.81 |
|  | 6 | 1.68 | 0.04 | 1.37 | 0.17 | 49.17 | 12.64 | 24.09 |
|  | 7 | 1.72 | 0.03 | 1.31 | 0.17 | 54.13 | 15.06 | 22.78 |
|  | 8 | 1.69 | 0.03 | 1.37 | 0.17 | 50.25 | 11.18 | 16.94 |
| Participant 2 | 5 | 1.48 | 0.06 | 1.51 | 0.27 | 92.41 | 36.84 | 28.35 |
|  | 6 | 1.73 | 0.13 | 1.89 | 0.26 | 93.45 | 28.71 | 25.37 |
|  | 7 | 1.66 | 0.12 | 1.75 | 0.27 | 92.50 | 36.82 | 34.99 |
|  | 8 | 1.59 | 0.10 | 1.92 | 0.27 | 103.22 | 61.42 | 54.48 |

Supplementary Table 1. Range of rigid body values across each recording. Does not include datapoints interpolated using the pchip function in Matlab (i.e. those where more than three markers were missing which were longer than 0.2 s) and so is an underestimate of the forward-back movement for Participant 1’s initial recording. The speed is estimated from the average Euclidean distance moved between recorded datapoints.


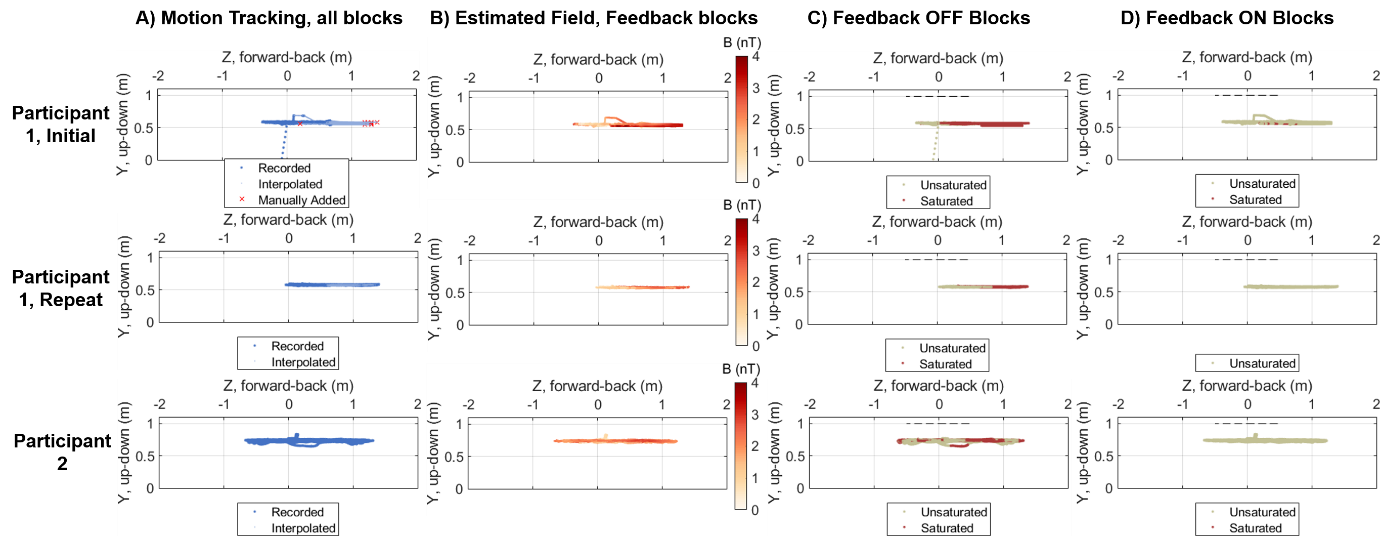


Supplementary Figure 5. Repeat of Figure 5 from the main manuscript, showing the trajectory of the participant through the room in the Y-Z plane. A) Position as recorded with OptiTrack Flex 13 motion tracking cameras in every walking block overlaid. Shows where motion tracking was successful (dark blue points), where data was manually added (red crosses) and where motion capture data was interpolated (light blue points). B) Background magnetic field magnitude over the participant’s path in feedback-walking blocks, estimated from the intended feedback applied. The field is approximately 3.5 nT at the front and right of the MSR but is below 1 nT in the centre. C) and D) Positions where at least 1 OPM channel was saturated (red) when feedback is off (C) and when feedback is on (D). A black, dashed circle marks a 50 cm radius from the centre of the room. The centre of the room is at coordinate (0,0,0).

## Comparison between Y and Z OPM channels


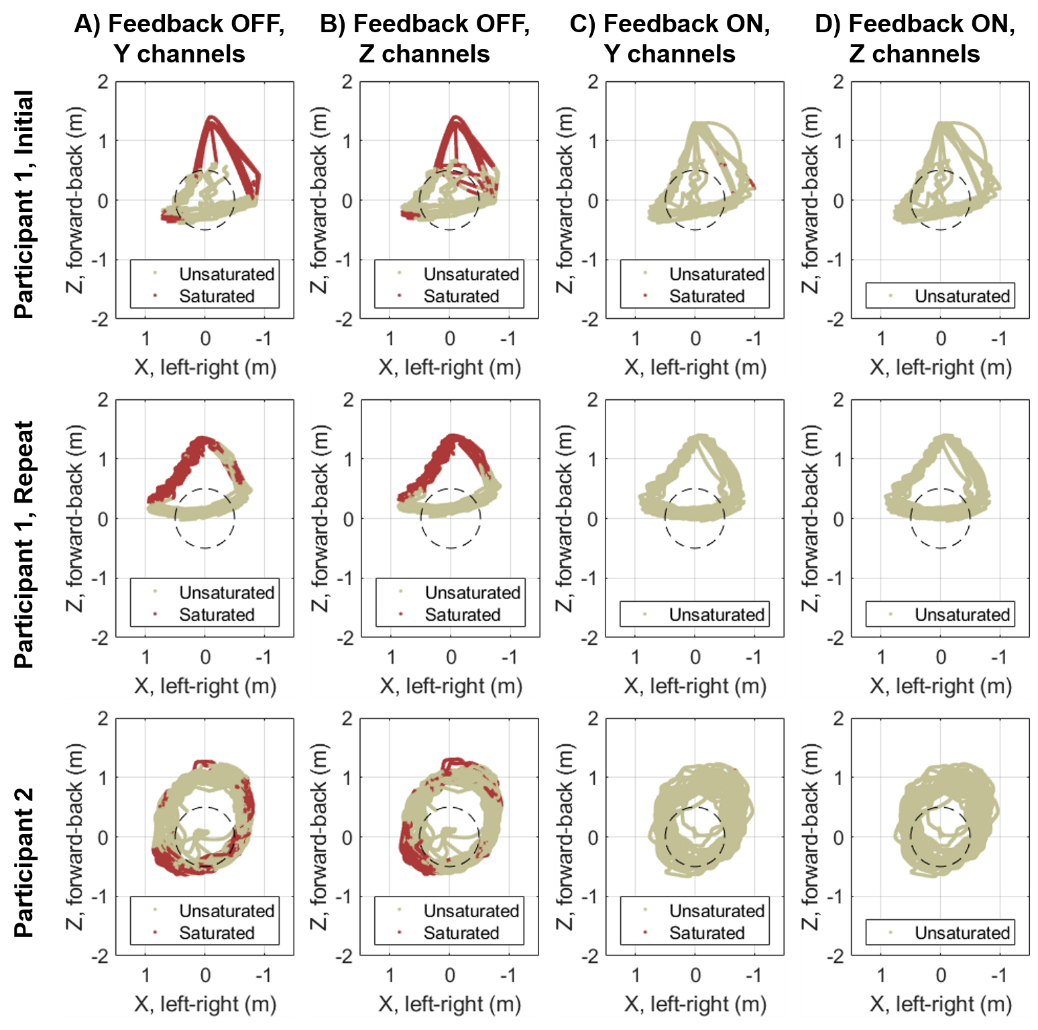


Supplementary Figure 6. Comparison of where in the MSR Y and Z OPM channels (i.e. channels radial to the head (Y) or tangential to it (Z)) saturated for each recording. A) and C) Y channels. B) and D) Z channels. A) and B) Feedback off, C) and D) feedback on. At the red positions, at least one OPM channel (of the given orientation) was saturated. A black, dashed circle marks a 50 cm radius from the centre of the MSR ((0,0,0)).

| Channel Name (OPM – channel orientation) | Number of Saturated Trials (out of 1120) | | | |
| --- | --- | --- | --- | --- |
|  | Participant 1 | | Participant 2 | |
|  | Feedback OFF | Feedback ON | Feedback OFF | Feedback ON |
| DU-Y | 1 | 0 | 115 | 0 |
| DU-Z | 267 | 0 | 20 | 0 |
| MV-Y | 0 | 0 | 18 | 0 |
| MV-Z | 178 | 0 | 111 | 0 |
| DL-Y | 24 | 0 | 98 | 2 |
| DL-Z | 304 | 0 | 105 | 0 |
| 1C-Y | 0 | 0 | 17 | 0 |
| 1C-Z | 352 | 0 | 108 | 0 |
| OH-Y | 3 | 0 | 61 | 0 |
| OH-Z | 271 | 0 | 7 | 0 |
| N0-Y | 104 | 0 | 31 | 0 |
| N0-Z | 294 | 0 | 21 | 0 |
| MT-Y | 0 | 0 | 74 | 0 |
| MT-Z | 182 | 0 | 4 | 0 |
| A7-Y | 303 | 0 | 8 | 0 |
| A7-Z | 365 | 0 | 44 | 0 |
| DR-Z | 244 | 0 | 18 | 0 |
| DR-Y | 9 | 0 | 83 | 0 |
| N4-Y | 191 | 0 | 41 | 0 |
| N4-Z | 180 | 0 | 42 | 0 |
| MZ-Y | 5 | 0 | 17 | 0 |
| MZ-Z | 172 | 0 | 102 | 0 |
| N3-Y | 6 | 0 | 121 | 0 |
| N3-Z | 249 | 0 | 66 | 0 |
| 17-Y | 10 | 0 | 216 | 0 |
| 17-Z | 259 | 0 | 106 | 0 |
| DQ-Y | 3 | 0 | 68 | 0 |
| DQ-Z | 265 | 0 | 17 | 0 |
| DO-Y | 135 | 0 | 38 | 0 |
| DO-Z | 0 | 0 | 0 | 0 |
| A3-Y | 4 | 0 | 25 | 0 |
| A3-Z | 259 | 0 | 109 | 0 |
| A9-Y | 0 | 0 | 11 | 0 |
| A9-Z | 205 | 0 | 12 | 0 |

Supplementary Table 2. Number of saturated trials in the walking conditions for each sensor when feedback is or is not used. Blocks of the same condition have been combined, giving 1120 total trials in each condition. Participant 1’s initial recording is not shown as a different selection of OPMs was used.


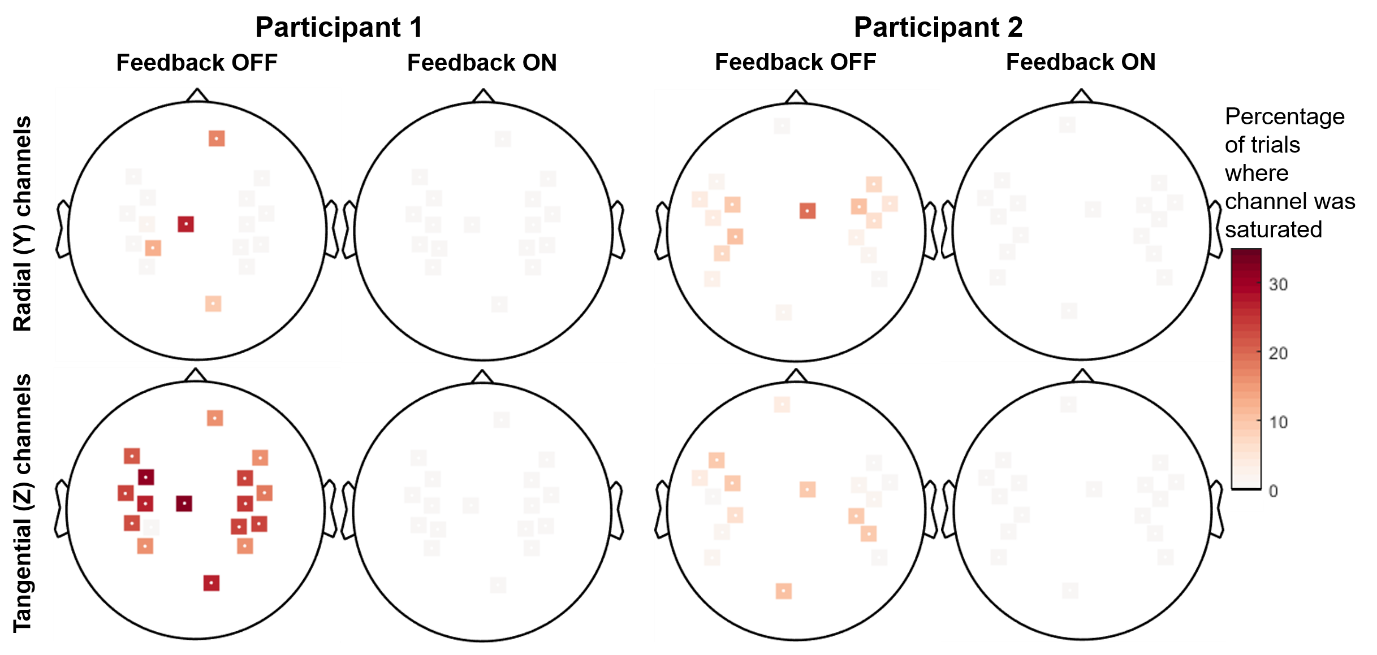


Supplementary Figure 7. Visual representation of Supplementary Table 1. Percentage of trials where each channel was saturated. For participant 1, more of the Z channels saturated more frequently than the Y channels. The Y channels around the auditory cortices were all orientated approximately along the left-right axis of the MSR. This suggests that the field in this direction does not vary as greatly with position as in the up-down (Y channel on the top of the head) or forward-back (Z channels) direction. The percentage of trials for which each channel saturated is more consistent across sensors for participant 2, possibly because they rotated their head by a greater degree during the experiment.

## Participant 1 Initial Recording

Due to the methodological differences between participant 1’s initial recording (stimulus delivered monaurally, sensor placement asymmetrical with poor coverage over the left hemisphere and only the walking condition included) and the recordings in the main manuscript, the results are presented slightly differently. Rather than a response at 100 ms, we observed an earlier M100 response at $\sim$ 92 ms in the left hemisphere and a later M100 response at $\sim$ 110 ms originating from the right hemisphere. Previous literature on auditory evoked potentials from monaural stimulation is somewhat inconclusive as to latency differences between hemispheres, but an earlier, larger amplitude response in the contralateral hemisphere (in this case left) has been frequently observed (Butler et al., 2009; Majkowski et al., 1971; Rothenberger et al., 1982).

Both when feedback is on and off, the 92 ms response appears to originate from or close to the left auditory cortex. The t-values are higher when feedback was used and there are more supra-threshold vertices. The 92 ms response has considerably larger t-values than the later 110 ms response, perhaps consistent with the previous literature, but it is difficult to confidently conclude such given the limited sensor coverage over the left hemisphere. At 110 ms, when feedback was used, the t-values are considerably smaller than the earlier 92 ms response but are consistent with a response from the right auditory cortex. The peak t-value lies within the right Rolandic operculum, adjacent to the right primary auditory cortex based on the AAL atlas (Tzourio-Mazoyer et al., 2002) while when feedback is off, the peak t-value is statistically insignificant and lies within the left supplementary motor area.


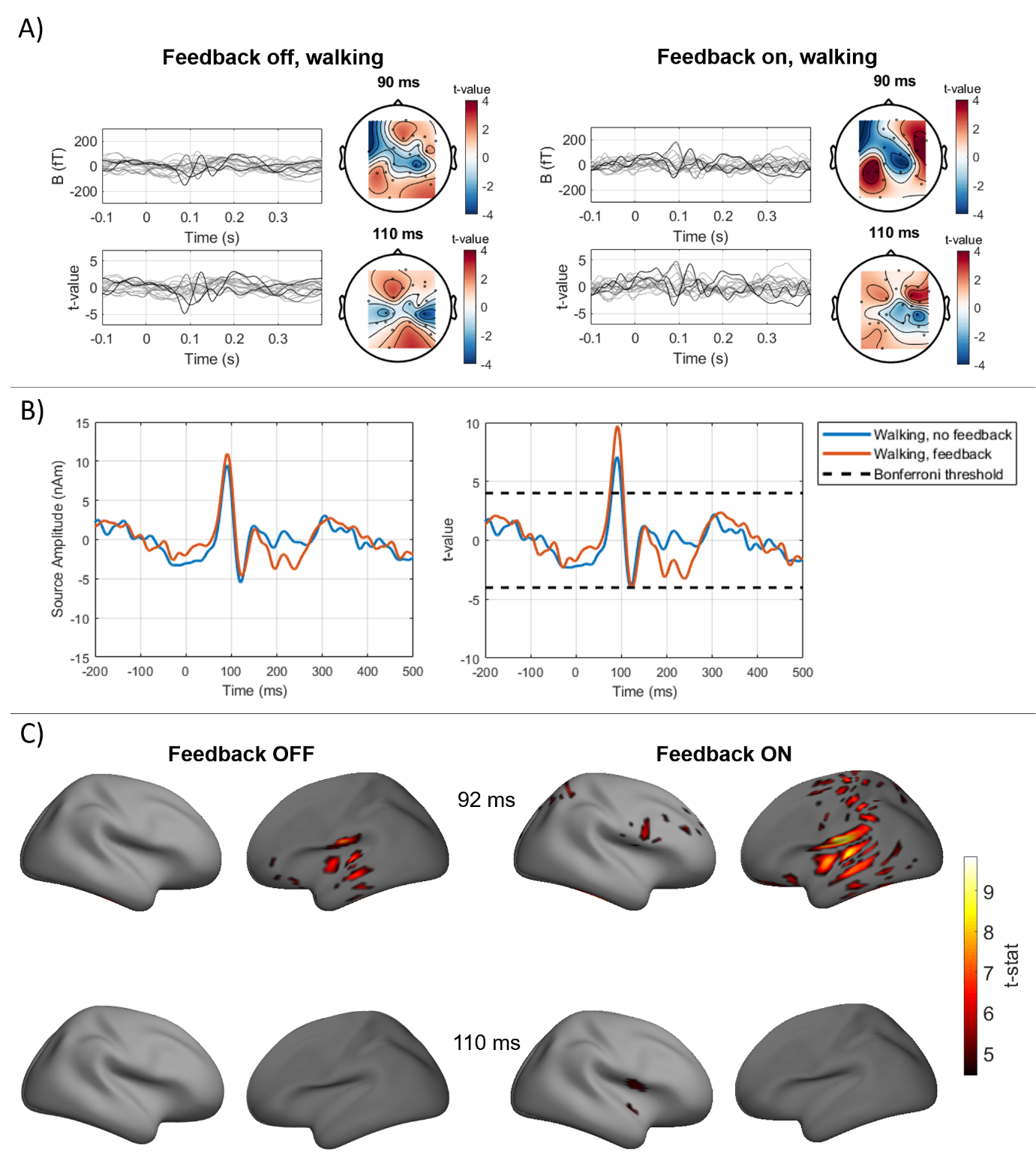


Supplementary Figure 8. Auditory evoked responses for participant 1’s initial recording. A) Sensor level auditory evoked response. Left, butterfly plot of all sensors, with colour indicating distance from auditory cortices (black closest, light grey furthest). Right, topography of response between (top) 85 ms and 95 ms and (bottom) 105 ms and 115 ms only for OPM channels radial to the head (Y channels). Left, feedback off; right, feedback on. B) Reconstructed evoked response waveforms from the left auditory cortex for the feedback off and on recordings. The significance threshold after Bonferroni correction for multiple comparisons is indicated with a dashed black line. C) T-statistic maps at 92 ms and 110 ms when feedback is off (left) and on (right). The earlier 92 ms map shows a response in and near the left auditory cortex. The later 110 ms map shows a response in the right auditory cortex for the feedback on case but there were no significant regions when feedback was off.

## Movement Regression

Linear regression of movement from OP-MEG recordings has previously been shown to reduce the movement related noise within the recordings. Here we have tested regressing movement out of the OPM recordings as in (Seymour et al., 2022), regressing the position and rotation of the rigid body recorded with the OptiTrack camera system from the OPM data over a 10 s long moving window. We did not include this pre-processing step in the main manuscript as it generally had no or a detrimental effect on the OPM recordings. We believe that this is due to the high degree of saturated OPM data during movement when feedback was not used, and because the feedback introduced was expected to remove the relationship between head position and rotation and the OPM recordings. When the participants were seated, there was very little movement to regress out. Following movement regression, the same pre-processing steps as in the main manuscript (offline HFC and filtering) were applied.


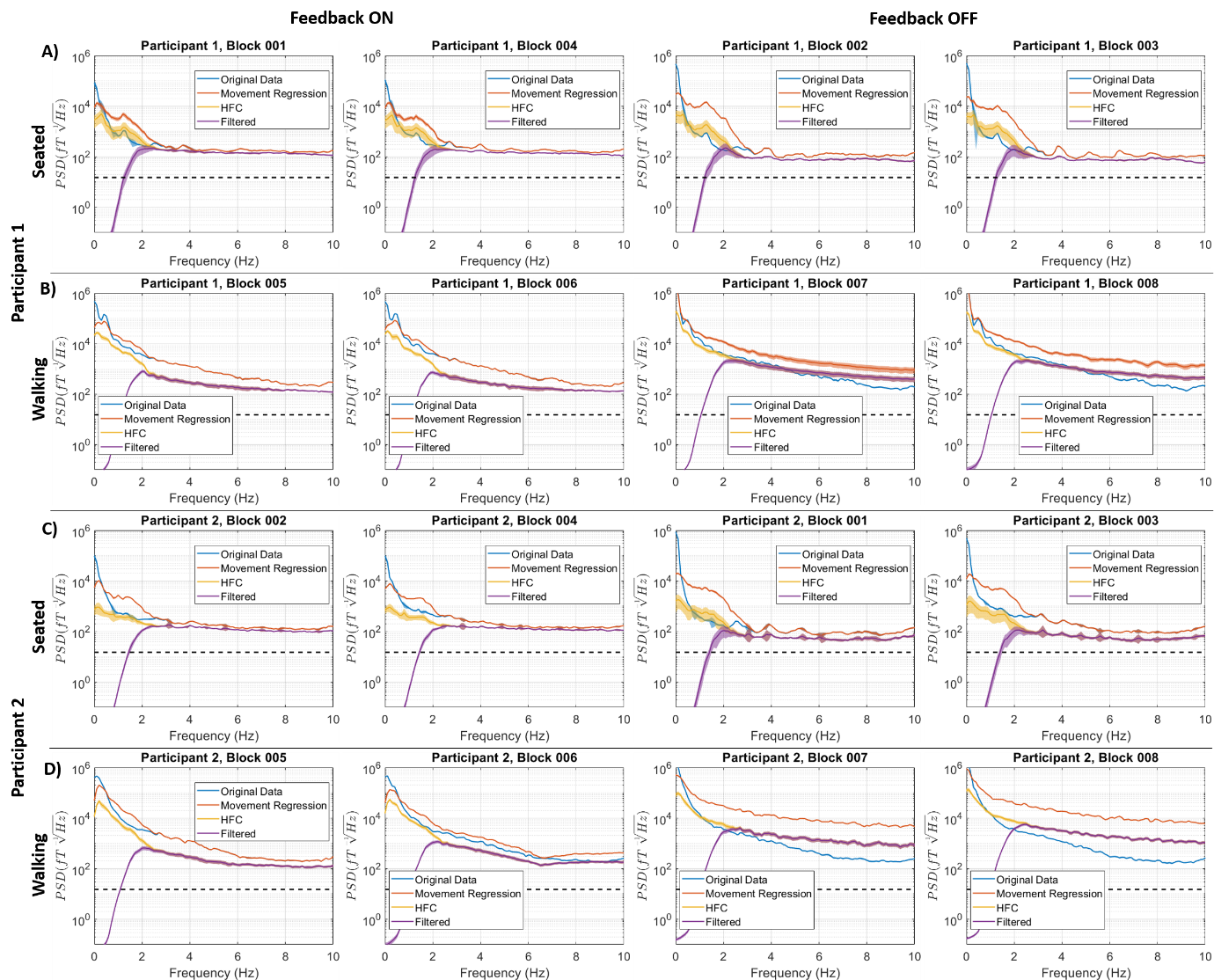


Supplementary Figure 9. Power spectral density plots of median OPM channel after each pre-processing step when movement regression is included. Movement regression was applied first, followed by HFC and filtering. Width of each line is the standard error of the median over channels. Feedback on blocks are on the left and feedback off blocks on the right. A) Participant 1, seated. B) Participant 1, walking. C) Participant 2, seated. D) Participant 2, walking. Movement regression is generally detrimental without feedback when walking as the OPM data are largely saturated and so effectively meaningless.


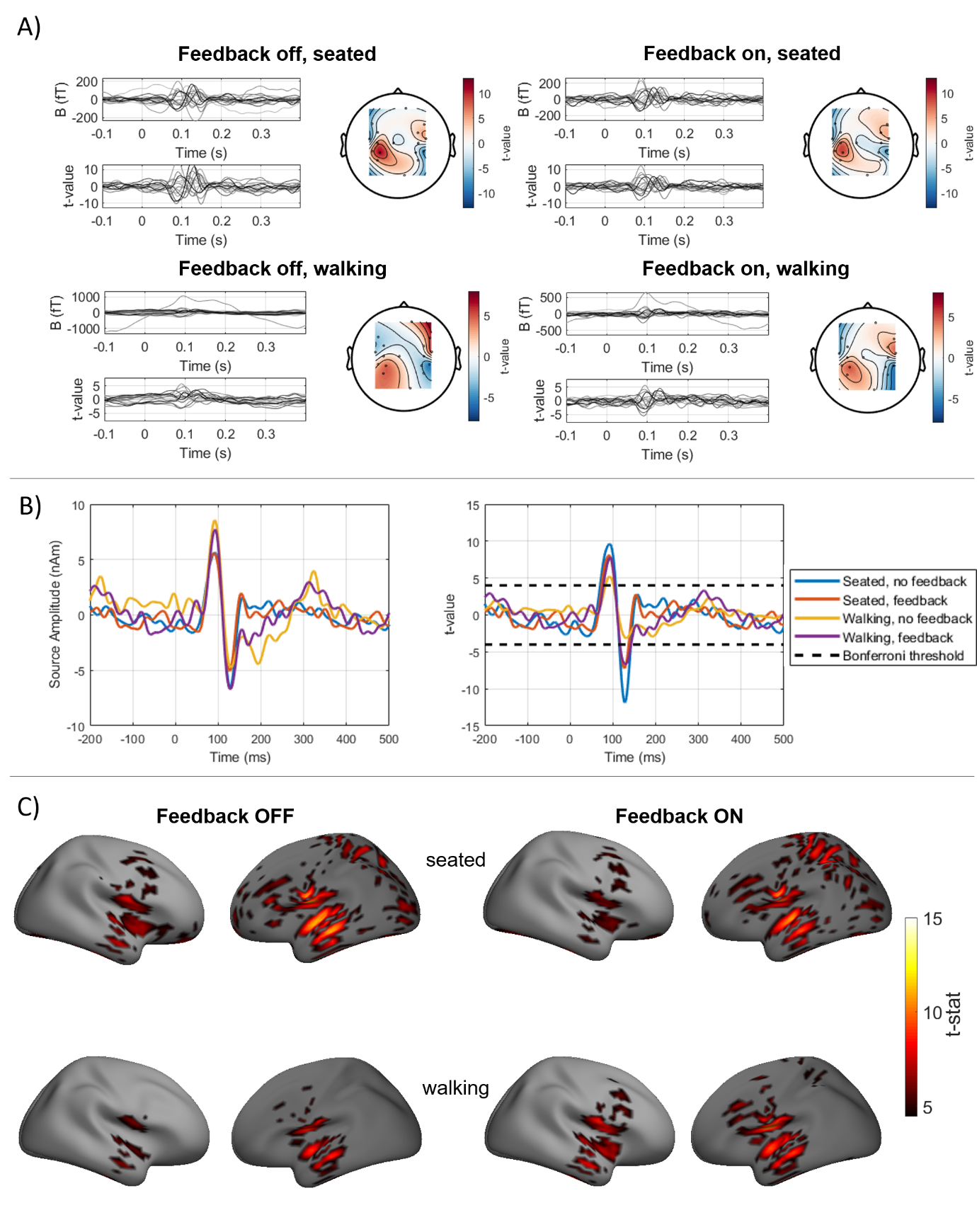


Supplementary Figure 10. Auditory evoked responses from Participant 1 when movement regression was included in the pre-processing pipeline. A) Sensor level auditory evoked response. Left, butterfly plot of all sensors with colour scaled by distance from auditory cortices (black closest, light grey furthest). Right, topography of response between 95 ms and 105 ms only for radial (Y) sensor channels. Left, feedback off; right, feedback on. Top, seated; bottom, walking. B) Reconstructed evoked response waveforms at the left auditory cortex for each condition. The significance threshold after Bonferroni correction for multiple comparisons is indicated with a dashed black line. C) T-statistic source maps at 100 ms when feedback is off (left) and on (right).

## Impact of offline HFC

Supplementary Figure 11, Supplementary Figure 12 and Supplementary Figure 13 look at the impact of applying HFC offline for each condition. Above 20 Hz, particularly in the seated case, offline HFC produces similar shielding factors whether feedback was applied (and hence real-time HFC was applied) or not. Since the real-time correction is low-pass filtered at 1 Hz, offline HFC is most effective above this frequency. HzHIt is noticeable that when feedback was on while walking, the 50 Hz and 100 Hz spectral peaks were widened in the raw data for both participant 1 and 2. Nevertheless, offline HFC reduces the width to that comparable with the feedback off case. Offline HFC has a smaller effect at low frequencies (< 10 Hz) when feedback was applied, as would be expected as it has effectively been applied in real-time in this region. It should also be noted that the very low frequency, feedback off – walking values are somewhat meaningless as it accounts for the saturated data. This likely explains the relatively low shielding factors from HFC in this region.


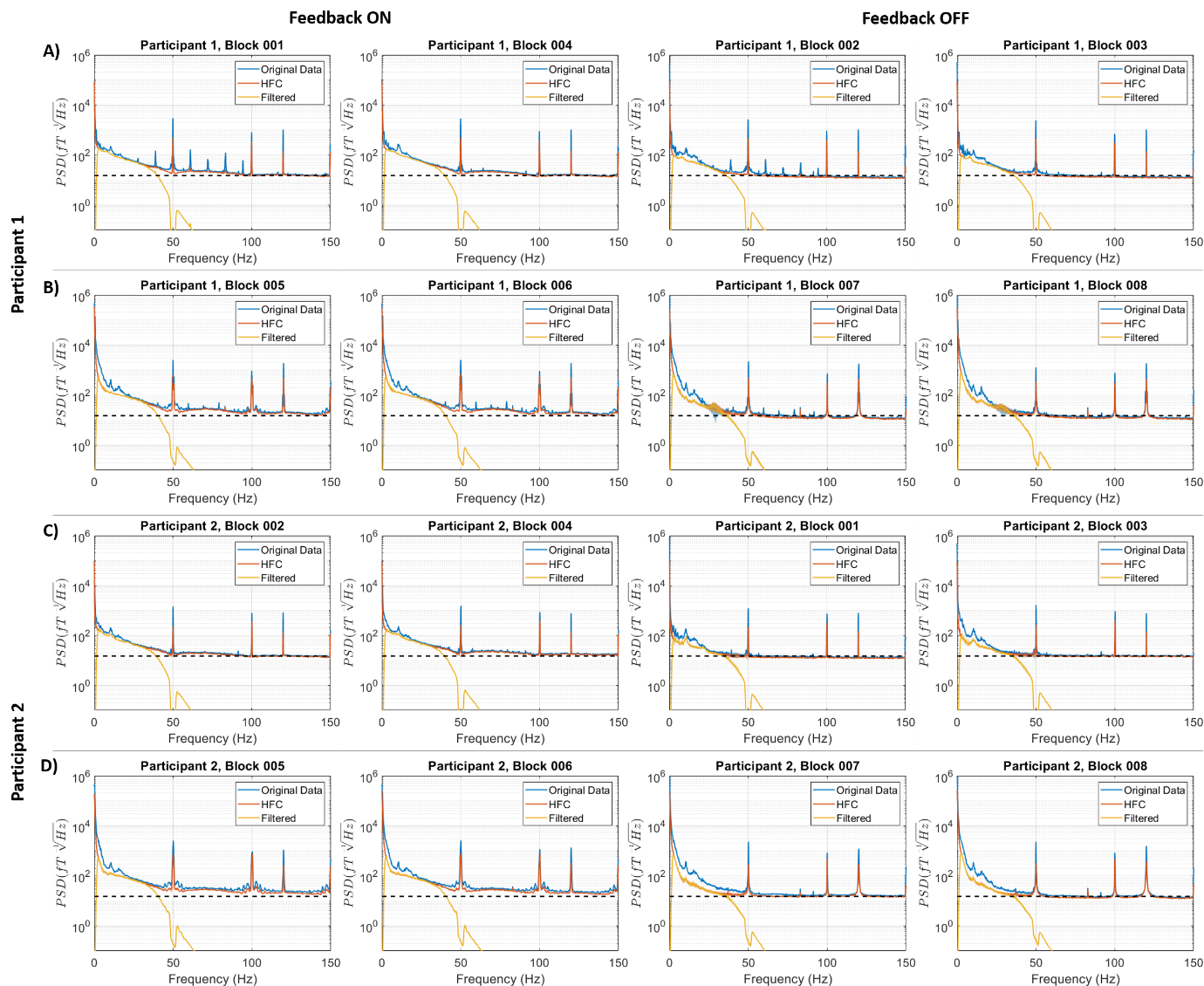


Supplementary Figure 11. The impact of HFC and filtering on the power spectra for each block. The median (over channels) power spectral density is shown after each processing step. The width is given by the standard error of the median over channels. A) participant 1, seated. B) participant 1, walking. C) participant 2, seated and D) participant 2, walking.


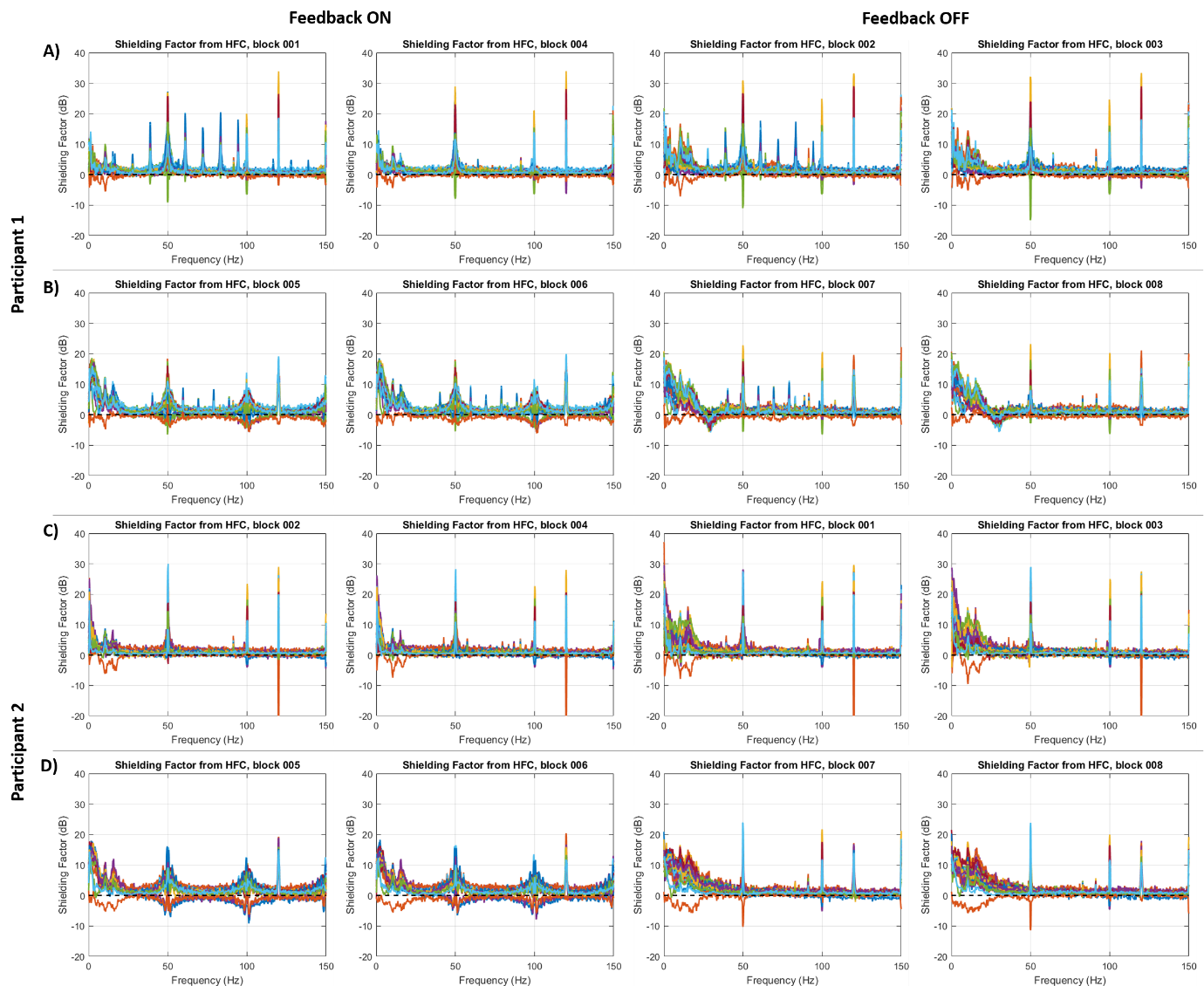


Supplementary Figure 12. The shielding factor from HFC for each channel and each block. Each coloured line is a different channel. A) participant 1, seated. B) participant 1, walking. C) participant 2, seated and D) participant 2, walking.


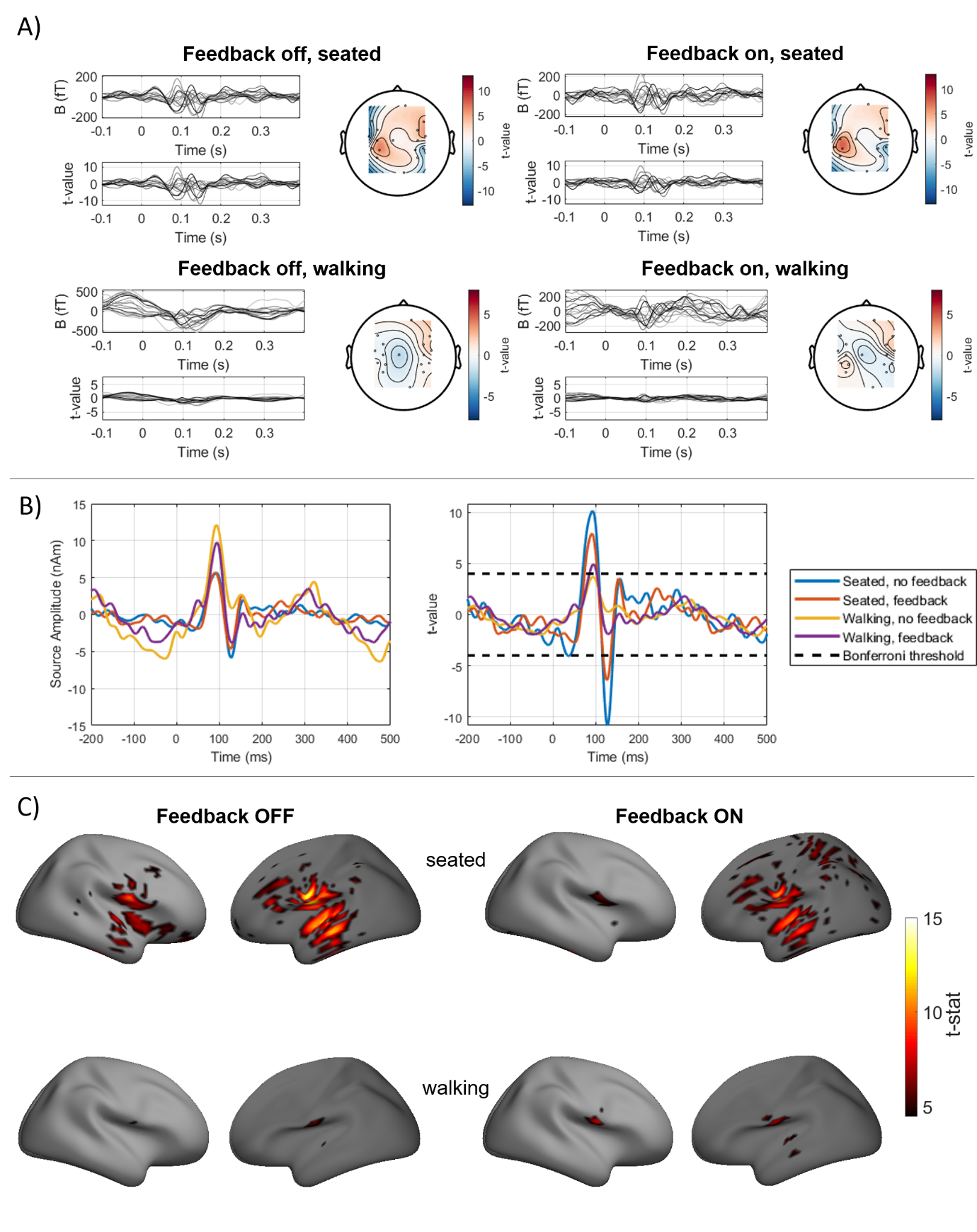


Supplementary Figure 13. Auditory evoked responses from participant 1 when HFC is not included offline in processing the OPM data. A) Sensor level auditory evoked response. Left, butterfly plot of all sensors with colour scaled by distance from auditory cortices (black closest, light grey furthest). Right, topography of response between 95 ms and 105 ms only for radial (Y) sensor channels. Left, feedback off; right, feedback on. Top, seated; bottom, walking. The sensor level t-values are considerably smaller than in the main manuscript where HFC is included. B) Reconstructed evoked response waveforms at the left auditory cortex for each condition. The significance threshold after Bonferroni correction for multiple comparisons is indicated with a dashed black line. C) T-statistic source maps at 100 ms when feedback is off (left) and on (right).

# External Coil Recordings

To further examine the dependence of feedback performance on the frequency of the external interference, we placed a set of external coils around the sensor array in the centre of the room. These coils were simple loops of wire on opposite walls on one side of the room, dimensions 2.2 m x 4 m, held up by hooks at each of the corners. The shape of the walls and their spacing meant that these were not Helmholtz coils, and so there will be some inhomogeneity in the magnetic field they produced across the OPM array. Nevertheless, they were successful in creating a varying external magnetic field in a controlled manner.

We recorded from the OPM array for 8 different coil frequencies: 0.1 Hz, 0.5 Hz, 1 Hz, 2 Hz, 4 Hz, 6 Hz, 8 Hz and 10 Hz. We repeated the recordings with the feedback off and on and did not include any additional filtering, beyond the implicit moving average filter introduced by the implementation of the model. The duration of the recordings was set to 10 wavelengths of the external interference. There were 26 OPMs, each recording from 2 axes and with feedback (when present) on all sensors. We also recorded what we had intended to output from LabView at each time point for each OPM channel.

To evaluate the performance of the feedback, we compared the recordings with feedback on and off, both looking at the time series and the shielding factor. Additionally, we looked at the difference between what we would expect to have recorded if we had fed-back what we were intending to and what was actually recorded.

## Time delay estimation

We found that there was likely to be a time delay between what we were intending to feedback and what was in fact fed-back to the sensor on-board coils. We inferred this as the amplitude of the feedback on recordings increased above the original feedback off amplitude when the frequency of the external interference was increased. To explain this further, it is known that the sum of two sinusoids is also a sinusoid

$$A_{1}\sin\left( \omega t+\phi_{1} \right)+A_{2}\sin\left( \omega t+\phi_{2} \right)=A_{3}\sin\left( \omega t+\phi_{3} \right)$$

Comparing this to the OPM recordings with feedback on during sinusoidal external interference, sine wave 1 is the external interference, wave 2 is what we fed-back and wave 3 is the consequent recording. Consider subtracting a sine wave from another sine wave, where they are perfectly out of phase but otherwise identical. The resulting amplitude ($A_{3}$) will be double that of the original sine wave ($A_{1}$) as the trough of one wave is subtracted from the peak of the other. We therefore sought to find the time delay between our intended feedback and the true feedback by determining the relationship between $A_{3}/A_{1}$ and the external interference frequency. Explicitly, when $A_{3}/A_{1}=2$, the phase difference $\phi_{2}-\phi_{1}$ equals $\pi$, and the time that corresponds to can be determined by $\pi/\omega$, so it is only necessary to determine the frequency $\omega$ at which $A_{3}/A_{1}=2$ to determine an expected time delay. This was a key motivation behind recording with multiple different interference frequencies.

## Results

Supplementary Figure 3 shows the time series for eight different frequencies of interest. The time series is shown with and without feedback. As could be anticipated from Figure 3 of the main manuscript, the feedback appears to work well for frequencies up to 2 Hz. However, above 4 Hz, the feedback distorts the external interference without noticeably reducing it. This implies that up to the 4 Hz sinusoid, the feedback is reducing the interference. Above this, the feedback is increasing the noise at each of the applied frequencies.


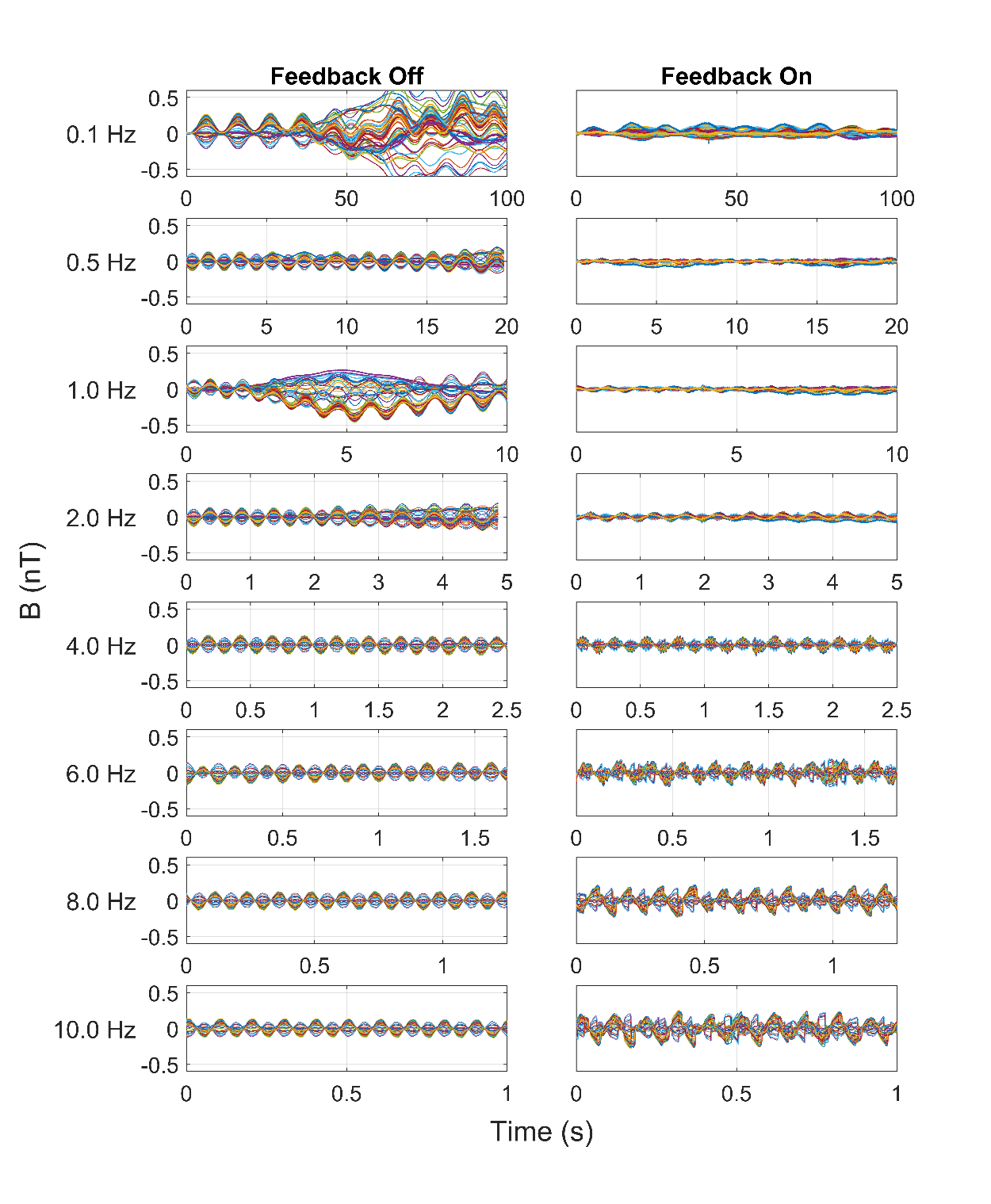


Supplementary Figure 14. Feedback off vs on for sinusoidal external interference of eight different frequencies. Each line corresponds to a different channel.

To test how well the feedback could perform if there were no time delays or added noise from applying the feedback, i.e. if what we intended to feedback was indeed what was fed-back, Supplementary Figure 4 shows the recording without feedback for a single channel, randomly selected, for a subset of the recorded frequencies (0.5 Hz, 4 Hz and 10 Hz), alongside the values which were intended to be fed back. Supplementary Figure 4 also shows the model subtracted out of the data and the recording with feedback. The theoretical performance is clearly higher than that in reality, showing that what we intended to feedback is not what is being fed-back. Looking at the 10 Hz interference in particular, introducing the feedback leads to both an increase in amplitude and an increase in apparent "spikiness" of the recording.

We suggest that the discrepancies between the ideal scenario and what was recorded could be at least partially explained by inaccuracies in the timing of the feedback system. We speculate that the spikiness is related to the time between updates to the feedback values. Supplementary Figure 5 tests this theory using the 10 Hz recording, by downsampling the intended feedback by increasing degrees, then interpolating to the nearest neighbour, to effectively simulate the case where the feedback is on at a single value for, for example, 50 ms, then steps to the next modelled value. The 10 ms and 50 ms cases in particular appears to recreate some of the spikiness of the recording with feedback on. Given the limitations of the serial control of the feedback currents, we know that there is a wait of at least 10 ms between one feedback update and the next, so this may explain some of the unusual behaviour observed.


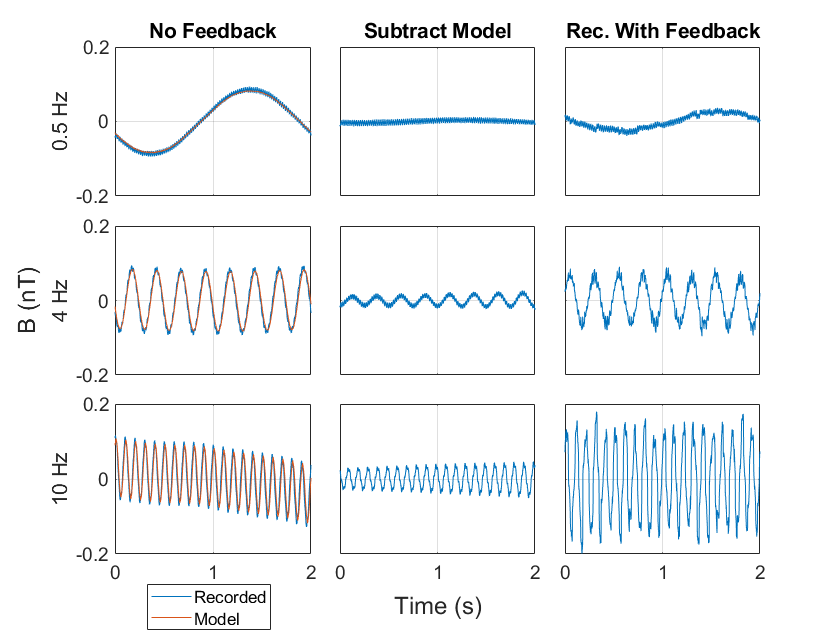


Supplementary Figure 15. The potential performance of the feedback for a single, randomly chosen channel. Each row is a different external coil frequency (0.5 Hz, 4 Hz or 10 Hz). The first column shows the recording without any feedback and what is intended to be fed back (the model). The middle column shows the difference between the recording and what was intended to be fedback, i.e. the best possible feedback performance. The right column is the true recording when, at a different time, the feedback was turned on for this channel with a sine wave of the given frequency produced by the external coils.

Considering the increase in amplitude between the ideal case and what we recorded, we speculated that this was due to a phase lag between the feedback and the input signal from the external coils. Supplementary Figure 6 shows the ratio of the amplitude of the interference with feedback on to the amplitude with feedback off for each of the different interference frequencies tested. Extrapolating these data via a linear fit, we expect that applying the feedback would double the amplitude of an 11.96 Hz interfering sinusoid. Consequently, as outlined in section 2.1, we found an expected time delay of 41.8 ms between the intended feedback and what was truly fed-back. The results of applying this to the intended feedback are shown in Supplementary Figure 7.

## Discussion


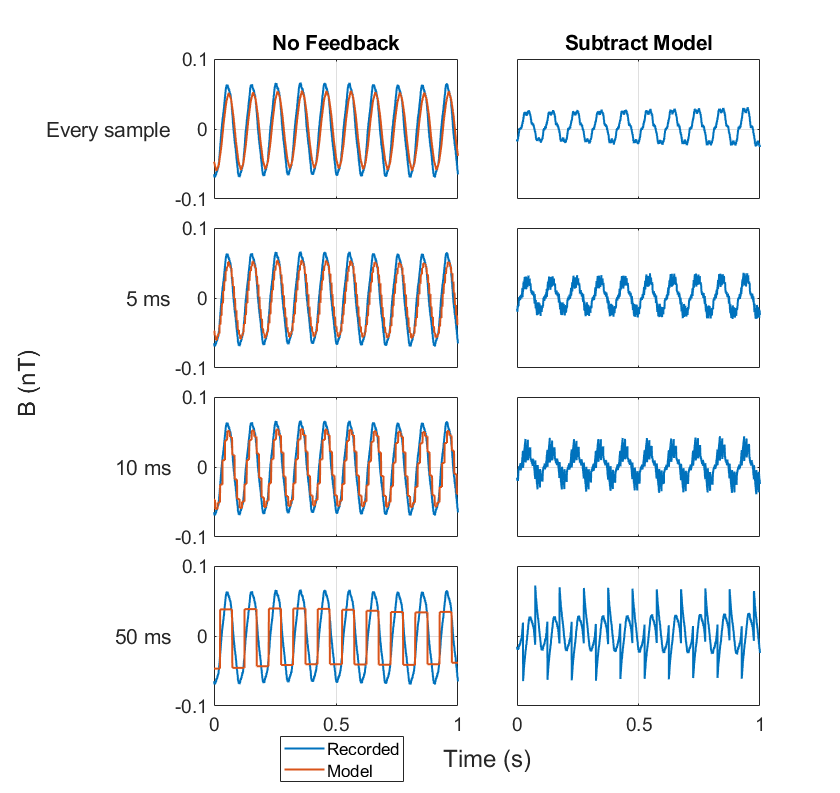


Supplementary Figure 16. Impact of updating feedback at discreet intervals. Repeat of the 10 Hz case in Supplementary Figure 2, except that the intended feedback is held constant for 1 sample, 5 ms, 10 ms and 50 ms.

We observed that the feedback did not perform as well as would be expected, if we were indeed feeding back what we had intended to the OPMs. We were able to reproduce the performance of the empirical system by downsampling the intended feedback, to imitate the case where the feedback is only updated at set number of samples, and by introducing a delay between the recording and intended feedback of 41.8 ms. Minimising this delay in future would reduce the time when the background field at the OPMs deviates from zero, and so improve the closed loop bandwidth and linearity of the OPMs.


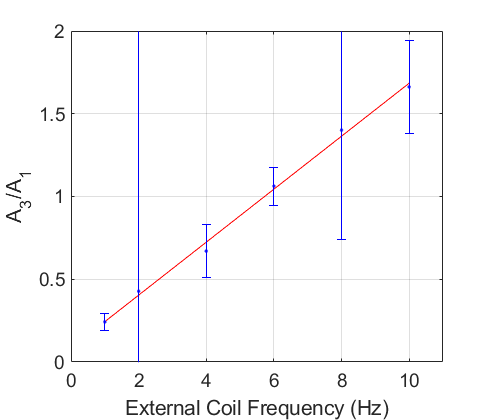


Supplementary Figure 17. Ratio of amplitude of signals with ($A_{3}$) and without feedback ($A_{1}$). Median value is shown by the point and errorbars given by the standard error of the mean across channels. Linear line of best fit also shown.


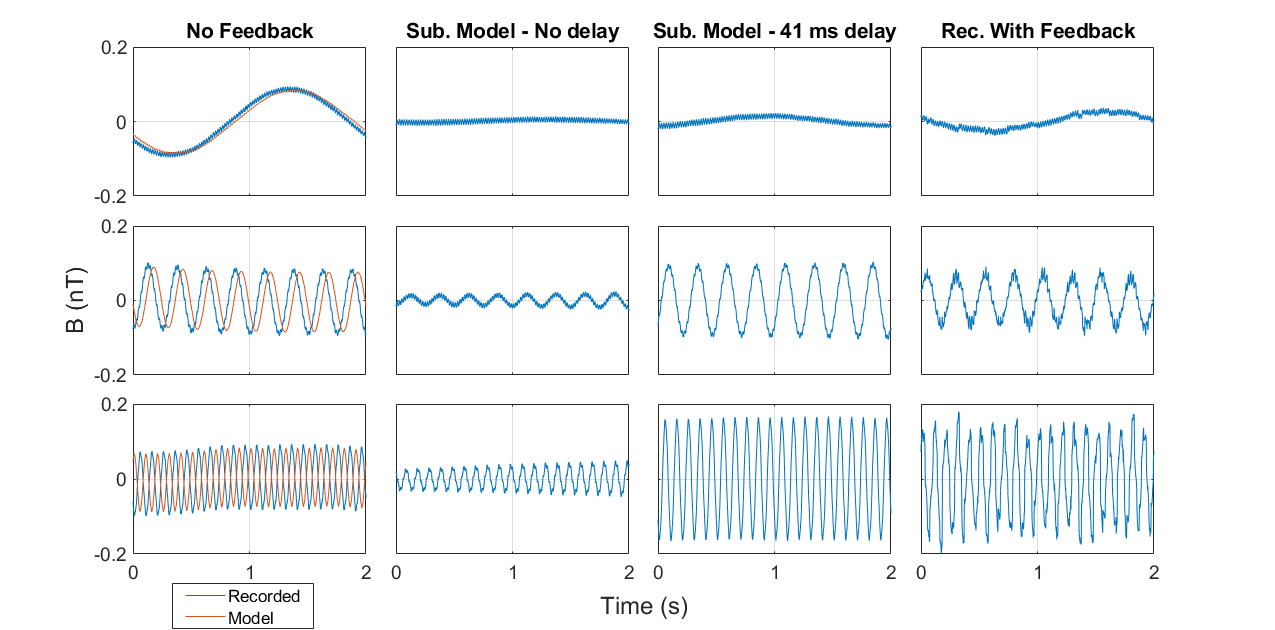


Supplementary Figure 18. Impact of introducing a lag to the intended feedback for interference of different frequencies (top row: 0.5 Hz, middle row: 4 Hz, bottom row: 10 Hz). Repeat of Supplementary Figure 3, except with a column added where the intended feedback (model) has been lagged behind the recording by 41.8 ms.

# References

Butler, R.A., Keidel, W.D., Spreng, M., 2009. An Investigation of the Human Cortical Evoked Potential Under Conditions Of Monaural and Binaural Stimulation. Acta Oto-Laryngologica. https://doi.org/10.3109/00016486909121570

Majkowski, J., Bochenek, Z., Bochenek, W., Knapik-Fijałkowska, D., Kopeć, J., 1971. Latency of averaged evoked potentials to contralateral and ipsilateral auditory stimulation in normal subjects. Brain Research 25, 416–419. https://doi.org/10.1016/0006-8993(71)90449-5

Rothenberger, A., Szirtes, J., Jürgens, R., 1982. Auditory evoked potentials to verbal stimuli in healthy, aphasic, and right hemisphere damaged subjects. Arch Psychiatr Nervenkr 231, 155–170. https://doi.org/10.1007/BF00343837

Seymour, R.A., Alexander, N., Mellor, S., O’Neill, G.C., Tierney, T.M., Barnes, G.R., Maguire, E.A., 2022. Interference suppression techniques for OPM-based MEG: Opportunities and challenges. NeuroImage 247, 118834. https://doi.org/10.1016/j.neuroimage.2021.118834

Tzourio-Mazoyer, N., Landeau, B., Papathanassiou, D., Crivello, F., Etard, O., Delcroix, N., Mazoyer, B., Joliot, M., 2002. Automated Anatomical Labeling of Activations in SPM Using a Macroscopic Anatomical Parcellation of the MNI MRI Single-Subject Brain. NeuroImage 15, 273–289. https://doi.org/10.1006/nimg.2001.0978
